# Ginsenoside Rb1 improves intestinal aging *via* regulating the expression of sirtuins in the intestinal epithelium and modulating the gut microbiota of mice

**DOI:** 10.3389/fphar.2022.991597

**Published:** 2022-09-27

**Authors:** Zili Lei, Lei Chen, Qing Hu, Yanhong Yang, Fengxue Tong, Keying Li, Ting Lin, Ya Nie, Hedong Rong, Siping Yu, Qi Song, Jiao Guo

**Affiliations:** ^1^ Guangdong Metabolic Diseases Research Center of Integrated Chinese and Western Medicine, Guangdong Pharmaceutical University, Guangzhou, China; ^2^ The First Affiliated Hospital (School of Clinical Medicine), Guangdong Pharmaceutical University, Guangzhou, China; ^3^ School of Traditional Chinese Medicine, Guangdong Pharmaceutical University, Guangzhou Higher Education Mega Center, Guangzhou, China

**Keywords:** Ginsenoside Rb1, gut microbiota, intestinal aging, sirtuin, intestinal integrity

## Abstract

Intestinal aging seriously affects the absorption of nutrients of the aged people. Ginsenoside Rb1 (GRb1) which has multiple functions on treating gastrointestinal disorders is one of the important ingredients from Ginseng, the famous herb in tradition Chinese medicine. However, it is still unclear if GRb1 could improve intestinal aging. To investigate the function and mechanism of GRb1 on improving intestinal aging, GRb1 was administrated to 104-week-old C57BL/6 mice for 6 weeks. The jejunum, colon and feces were collected for morphology, histology, gene expression and gut microbiota tests using H&E staining, X-gal staining, qPCR, Western blot, immunofluorescence staining, and 16S rDNA sequencing technologies. The numbers of cells reduced and the accumulation of senescent cells increased in the intestinal crypts of old mice, and administration of GRb1 could reverse them. The protein levels of CLDN 2, 3, 7, and 15 were all decreased in the jejunum of old mice, and administration of GRb1 could significantly increase them. The expression levels of *Tert*, *Lgr5*, *mKi67*, and *c-Myc* were all significantly reduced in the small intestines of old mice, and GRb1 significantly increased them at transcriptional or posttranscriptional levels. The protein levels of SIRT1, SIRT3, and SIRT6 were all reduced in the jejunum of old mice, and GRb1 could increase the protein levels of them. The 16S rDNA sequencing results demonstrated the dysbiosis of the gut microbiota of old mice, and GRb1 changed the composition and functions of the gut microbiota in the old mice. In conclusion, GRb1 could improve the intestinal aging *via* regulating the expression of Sirtuins family and modulating the gut microbiota in the aged mice.

## Introduction

The growing of aging societies is one of the major challenges for today’s medical science (Friedrich, 2019). The nutrients absorption ability of the intestines becomes impaired with age (Pénzes, 1984) and causes the vulnerability to disease and the physical weakness of the elderly peoples (Ben Othman et al., 2020). It was also found that the morphology of jejunum changed in old rats (Hassan et al., 2017). Therefore, it would be meaningful to develop drugs for improving intestinal aging.

Ginsenoside Rb1 (GRb1) is the important ingredient from *Panax ginseng* Meyer which is the famous herb in traditional Chinese medicine (Lin et al., 2022). The *Panax ginseng* has been widely used to treat many kinds of disease. Recent study showed that the doxorubicin-induced early cancer therapeutics-related cardiac dysfunction and early decline in left ventricular ejection fraction in breast cancer patients can be protected through prophylactic Panax ginseng supplementation (Hamidian et al., 2022). The lifespan of *Drosophila* is extended with the treatment of total ginsenosides (TGGR), the main active components in Panax ginseng (Zhao et al., 2022b). Many types of ginsenosides have been demonstrated to have neuroprotective effects (Zhao et al., 2022a). There are around 200 ginsenosides have been detected from ginseng and GRb1 is one type of major ginsenosides (Zhao et al., 2022a; Hyun et al., 2022).

GRb1 has been reported to have multiple functions in various diseases. It can be used to treat obesity, hyperglycemia and diabetes through multi-targets (Zhou et al., 2019; Xiong et al., 2010). GRb1 also can ameliorate diabetic kidney podocyte injury *via* inhibiting the activity of aldose reductase (He et al., 2022). It was also found that GRb1 can reduce the myocardial ischemia/reperfusion injury *via* inhibiting cardiomyocyte autophagy through the PI3K/AKT/mTOR pathway (Qin et al., 2021). GRb1 also has anti-aging effect (Cheng et al., 2005), but the related mechanism is unclear. GRb1 can be used to treat many kinds of gastrointestinal disorders. It improves colitis in mice *via* alleviating endoplasmic reticulum (ER) stress through activating Hrd1 signaling pathway (Dong et al., 2021). GRb1 also can reduce ischemia/reperfusion-induced intestinal injury *via* activating PI3K/AKT/Nrf2 pathway (Chen et al., 2019). It was also found that GRb1 can promote the intestinal epithelial would healing of rats *via* activating ERK and Rho signaling (Toyokawa et al., 2019). GRb1 can protect the peritoneal air exposure caused intestinal mucosa damage in rats (Zhou et al., 2016). However, it is still unclear if GRb1can improve intestinal aging.

There are many genes have been reported to be related to the aging of intestines and other tissues. Stem cell exhaustion is one of the hallmarks of aging (López-Otín et al., 2013). Lgr5 is the mark gene of intestinal stem cells (Lei et al., 2012; Baghdadi et al., 2022). Telomerase plays important role in the intestinal stem cells and TERT is the important telomerase subunit (Hoffmeyer et al., 2012). Sirtuins, including Sirt1-7 in mammals, have been demonstrated to play important roles in maintaining the longevity of various tissues (Gámez-García and Vazquez, 2021; Yang et al., 2021a; Watroba and Szukiewicz, 2021). Hence, it would be very meaningful to explore if GRb1 could regulate the expression of these genes in the intestines of aging mice.

Many studies have demonstrated the changes of the composition and functions of gut microbiota with aging (Ishaq et al., 2021; Niu et al., 2021; Ruiz-Gonzalez et al., 2022). It was reported that specific bacterial community pattern and signature taxa are related to longevity of people (Ren et al., 2021). The dysbiosis of gut microbiota is also associated with age-related disorders (Sharma, 2022). Relationships between gut microbiota and age-related macular degeneration have been found (Lima-Fontes et al., 2021). Gut microbiota-derived pro-inflammatory neurotoxins have been detected in brain cells and tissues of aged people with Alzheimer’s disease (Lukiw et al., 2021; Zhao et al., 2021). Gut microbiota dysbiosis has also been found to promote the age-related atrial fibrillation *via* activating NLRP3-inflammasome (Zhang et al., 2021). GRb1 can improve glucose and lipid metabolic disorders through regulating gut microbiota of high fat diet induced obesity mice (Yang et al., 2021b; Bai et al., 2021). GRb1 also can be converted into compound K by the gut microbiota to prevent inflammatory-associated colorectal cancer (Yao et al., 2018). However, it still needs to explore whether GRb1 could improve intestinal aging *via* modulating gut microbiota.

In the present study, we reported the function and mechanisms of GRb1 on improving the intestinal aging of old mice. Our work encouraged the exploration of drugs for prevention and treatment of age-related diseases.

## Materials and methods

### Mice

**FIGURE 1:**
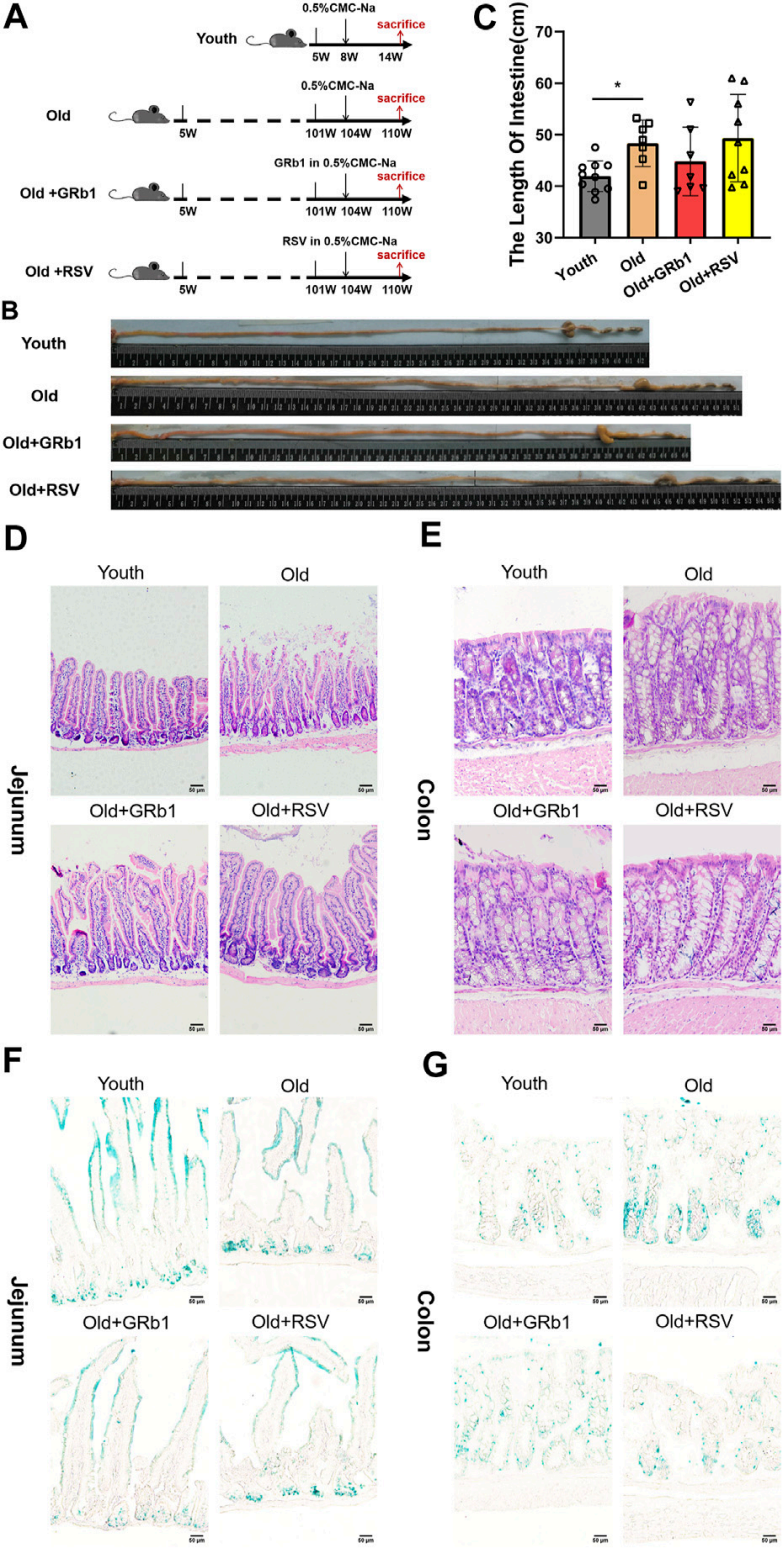
GRb1 showed the potential of improving intestinal aging of old mice. **(A)** The scheme of the experimental design. **(B)** Representative images of the intestines of mice from Youth, Old, Old + GRb1 and Old + RSV groups. **(C)** The length of intestines from each group. **(D,E)** Representative images of **(D)** jejunal and **(E)** colonal sections of H&E staining. **(F,G)** Representative images of **(F)** jejunal and **(E)** colonal sections of beta-galactosidase staining. ^*^ *p* < 0.05 compared with the Youth group. Scale bar, 50 μm. W, weeks of age; GRb1, Ginsenoside Rb1; RSV, Resveratrol.

All animal experimental procedures were approved by the Experimental Animal Ethics Committee of Guangdong Pharmaceutical University. Female C57BL/6 mice (5-week-old) purchased from Hunan Lex Jingda Laboratory Animal Co., Ltd. (Changsha, Hunan Province, China), were housed in the specific pathogen-free (SPF) animal facility, at 25°C, 60%–65% humidity, 12 h light-dark cycle, with free access to water and food. At the age of 104-week-old, the mice were randomly divided into three groups, 10 mice in each group. The Old + GRb1 group was administrated with GRb1 (50mg/kg; Meilunbio, Dalian, China; MB6856-1) intragastrically once a day. The GRb1 was diluted in 0.5% CarboxyMethylCellulose-Na (CMC-Na) (Tianjin Zhiyuan Chemical Reagent Co., Ltd., Tianjin, China). The Old group was administrated with the corresponding volume of 0.5% CMC-Na intragastrically once a day. Resveratrol (RSV; Meilunbio, Dalian, China; MB5267-1) was used as the positive drug. The Old + RSV group was intragastrically administrated with RSV (50 mg/kg) diluted in 0.5% CMC-Na once a day. The 8-week-old mice in Youth group was used as control, and they were also administrated with the corresponding volume of 0.5% CMC-Na intragastrically once a day. After 6 weeks of administration, the intestines were collected (Figure 1A).

### H&E staining and X-gal staining

The H&E staining was performed as previously (Lei et al., 2021b). Briefly, intestinal tissues were fixed in 4% paraformaldehyde at 4°C for overnight, then dehydrated, embedded in paraffin and sectioned. 4-µm-thick sections were stained with hematoxylin (H9627, Sigma-Aldrich) for 3 min, and then followed with eosin (E4009, Sigma-Aldrich) for 20 s at room temperature.

For X-gal staining, intestinal tissues were embedded in optimal cutting temperature compound (OCT) (Sakura Finetek) and sectioned. 7-µm-thick frozen sections were stained according to the manufacturer’s protocols for Senescence Detection Kit (Abcam, ab65351).

Images for H&E staining and X-gal staining were got using the Olympus DP74 microscope.

### Immunofluorescence staining

The immunofluorescence staining was performed as previously (Lei et al., 2021b). The intestinal tissues were fixed in 4% paraformaldehyde at 4°C for overnight, then dehydrated, embedded in OCT compound and sectioned. 7-µm-thick frozen sections were first boiled in 10 mM citric acid (Merck) at pH 6.0 for 5 min, then exposed in goat serum blocking buffer (ZSGB-BIO, ZLI- 9056) to block nonspecific sites for 1h at room temperature, following incubated with primary antibodies in blocking buffer at 4° C for overnight, and then with secondary antibodies for 1h at room temperature. The primary and secondary antibodies were listed in Supplementary Table 1. Images were got by using Olympus confocal microscope.

### qRT-PCR

Total RNA was extracted from each jejunal and colonal tissue using Trizol reagent (T9108, Takara Bio, Inc.), then subjected to reverse transcription *via* the PrimeScript™ RT Reagent kit (RR047A, Takara Bio, Inc.) at 37°C for 15 min and then 85°C for 5 s. The qPCR was conducted through the SYBR Premix Ex Taq kit (RR820A, Takara Bio, Inc.) *via* the LightCycler 480II System (Roche, Inc.). The processes of cycling were: 95°C for 30 s; followed 40 cycles of 95°C for 5 s, then 60°C for 20 s and 65°C for 15 s. Mouse GAPDH was used as the internal reference. All primers were listed in Supplementary Table 2.

### Western blot

Jejunal and colonal tissues of mice were lysed using the Radio-Immunoprecipitation Assay lysis buffer (MA0151, Dalian Meilun Biotechnology co., Ltd., Dalian, China), centrifuged at 13,680 x g, 4°C, for 30 min, then the supernatant was collected. Protein concentration was measured by the BCA kit (P0011, Beyotime, Shanghai, China). Equal amounts of protein (40 μg) were separated through the SDS-PAGE, subsequently transferred to a PVDF membrane. The PVDF membrane was blocked using the 5% skimmed milk (0040895, Biosharp, Hefei, China) in TBST buffer at room temperature for 1 h, incubated with primary antibodies in 4°C for overnight, and then incubated with HRP (horseradish peroxidase)-labeled secondary antibodies, the signals were detected *via* the enhanced chemiluminescence reagent. The primary and secondary antibodies were listed in Supplementary Table 3. The quantification of western blot bands was analyzed using the Lane 1d software (version 5.1.0.0; SageCreation).

### The 16S rRNA gene analysis

Fecal samples were quickly collected and frozen in the liquid nitrogen and stored at −80°C. The extraction of fecal bacterial DNA, PCR amplification of 16S rRNA genes, sequencing, and analysis were performed by the Gene *Denovo* Biotechnology Company (Guangzhou, China). The experimental procedures were performed as previously (Lei et al., 2021a).

### Statistical analysis

Statistical differences were determined *via* the SPSS software (version 25.0; IBM Corp.). Mean ± SE was used to express data. One-way ANOVA was performed between two groups. *p*-value<0.05 was considered to be significant.

## Results

### GRb1 improved the aging state of intestines of old mice

After 6 weeks of the administration of GRb1 or RSV, 7 (70%) mice survived in each of Old and Old + GRb1 groups, 9 (90%) mice survived in Old + RSV group, and all the mice survived in the Youth group. The intestines of old mice were significantly longer than the Youth group, and they are shorter but not significant in mice of the Old + GRb1 group compared to the Old group (Figures 1B,C). The numbers of cells in crypts of jejunum from old mice decreased compared to the Youth group, and it was increased after administration of GRb1 or RSV (Figure 1D). The numbers of cells in crypts of the colon of old mice were also lower than that of yang mice, and the administration of GRb1 or RSV could also improve it (Figure 1E).

The increase of cellular senescence is another hallmark of aging (López-Otín et al., 2013). Therefore, senescence-associated beta-galactosidase (X-gal) staining was next performed. The accumulation of senescent cells increased in crypts of jejunum from the Old group compared to young mice, and GRb1 or RSV could reduce them (Figure 1F; Supplementary Figure 1A). The senescence-associated signal was stronger in the colon of old mice than the Youth group, and it became weak and reduced after administration of GRb1 or RSV (Figure 1G; Supplementary Figure 1B). The intestinal stem and progenitor cells are localized in the crypts of the intestines. So, the increase of the numbers of the X-gal stained cells in the crypts of the intestines indicated the aging of the intestinal stem and progenitor cells of the old mice. Hence, the administration of GRb1 or RSV could improve the aging of the intestinal stem and progenitor cells of these mice. These results demonstrated that GRb1 could improve the aging state of intestines from old mice.

### GRb1 improved the intestinal integrity of old mice

**FIGURE 2:**
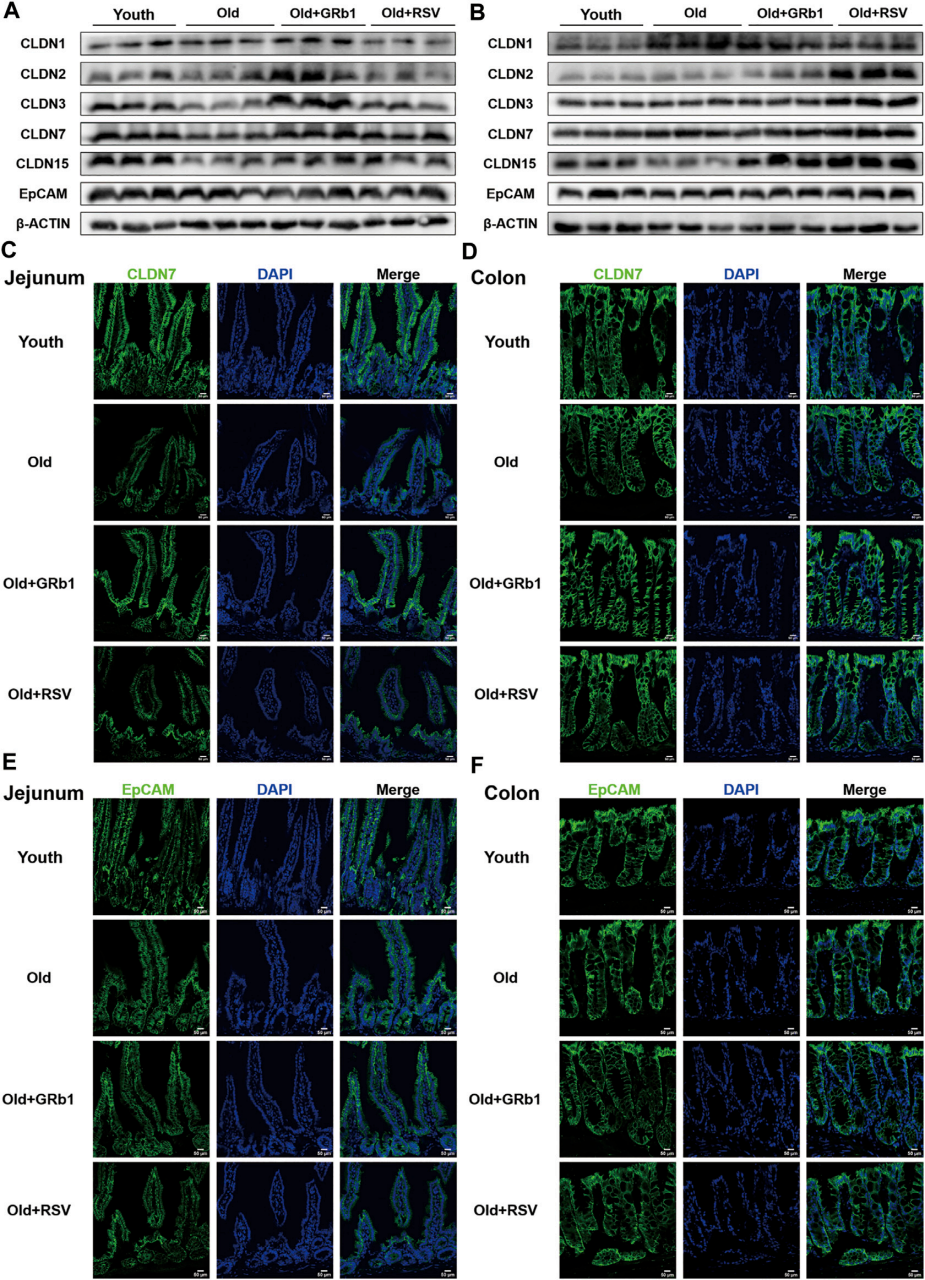
GRb1 improved the expression and localization of junctional proteins in the intestines of old mice. **(A,B)** Images of Western blot bands of CLDN 1, 2, 3, 7,15, and EpCAM in the **(A)** jejunum and **(B)** colon. **(C,D)** Representative images of immunofluorescence staining with antibodies to CLDN 7 of frozen sections of **(C)** jejunum and **(D)** colon. **(E,F)** Representative images of immunofluorescence staining with antibodies to EpCAM of frozen sections of **(E)** jejunum and **(F)** colon. Scale bar, 50 μm.

The increase of the permeability of the intestinal barrier has been reported in both aged human and animals (Tran and Greenwood-Van Meerveld, 2013; Parrish, 2017; Li et al., 2021), indicating the impaired intestinal integrity with aging. Hence, the protein levels of CLDN 1, 2, 3, 7, and 15 which are abundant components of tight junctions (TJs) in the intestinal epithelium (Lei et al., 2012; Lei et al., 2020) were first checked. CLDN 3, 7, and 15 were all significantly reduced in the jejunum of Old group compared to young mice, and the administration of GRb1 increased the expression of them (Figure 2A; Supplementary Figures 2C–E). CLDN 2 was also reduced in the jejunum of old mice, and it was also increased after GRb1 administration, although these changes were not significant (Figure 2A; Supplementary Figure 2B). RSV also could improve the expression of CLDN 3, 7, and 15, but the level of CLDN 2 had no significant change in the Old + RSV group (Figure 2A; Supplementary Figures 2B–E). Immunofluorescence staining results showed that the localization of CLDN 7 was still normal in the jejunum from Old group, but the expression level of it was significantly lower in Old group than the Youth, Old + GRb1 and Old + RSV groups (Figure 2C). The protein level of CLDN 1 had no significant difference in the jejunum of mice among the Youth, Old, Old + GRb1 and Old + RSV groups (Figure 2A; Supplementary Figure 2A). The protein level of EpCAM which is essential to maintain the functional tight junctions in the intestinal epithelium *via* recruiting proteins of Claudins (Lei et al., 2012; Wu et al., 2013) was significantly lower in the jejunum of Old group than the Youth group, and administration of GRb1 could not improve it (Figure 2A; Supplementary Figure 2F). The administration of RSV could significantly increase the protein level of EpCAM in the jejunum of old mice (Figure 2A; Supplementary Figure 2F). However, the localization of EpCAM had no significant difference in the jejunum of mice among the four groups (Figure 2E).

CLDN 15 was also lower in the colon of Old group than the Youth group, although the decrease was not significant (Figure 2B; Supplementary Figure 3E). The administration of GRb1 or RSV could significantly increase the protein level of CLDN 15 in the colon of old mice (Figure 2B; Supplementary Figure 3E). CLDN 1 and 2 were all increased in the colon of Old group compared to the Youth group, and CLDN 2 was significantly increased in the Old + GRb1 and Old + RSV groups (Figure 2B; Supplementary Figures 3A,B). CLDN 3 and 7 were all significantly increased in the colon of old mice compared to the Youth group, and RSV could also increase them in the colon of old mice but not significantly (Figure 2B; Supplementary Figures 3C, D). The immunofluorescence staining results confirmed that the localization of CLDN 7 had no significant difference in the colon among the four groups (Figure 2D). The expression and localization of EpCAM had no significant difference in the colon among the Youth, Old, Old + GRb1 and Old + RSV groups (Figures 2B,F; Supplementary Figure 3F). These results demonstrated that GRb1 could improve the integrity of intestinal epithelium of old mice.

### GRb1 improved the function of intestinal stem and progenitor cells of old mice

**FIGURE 3:**
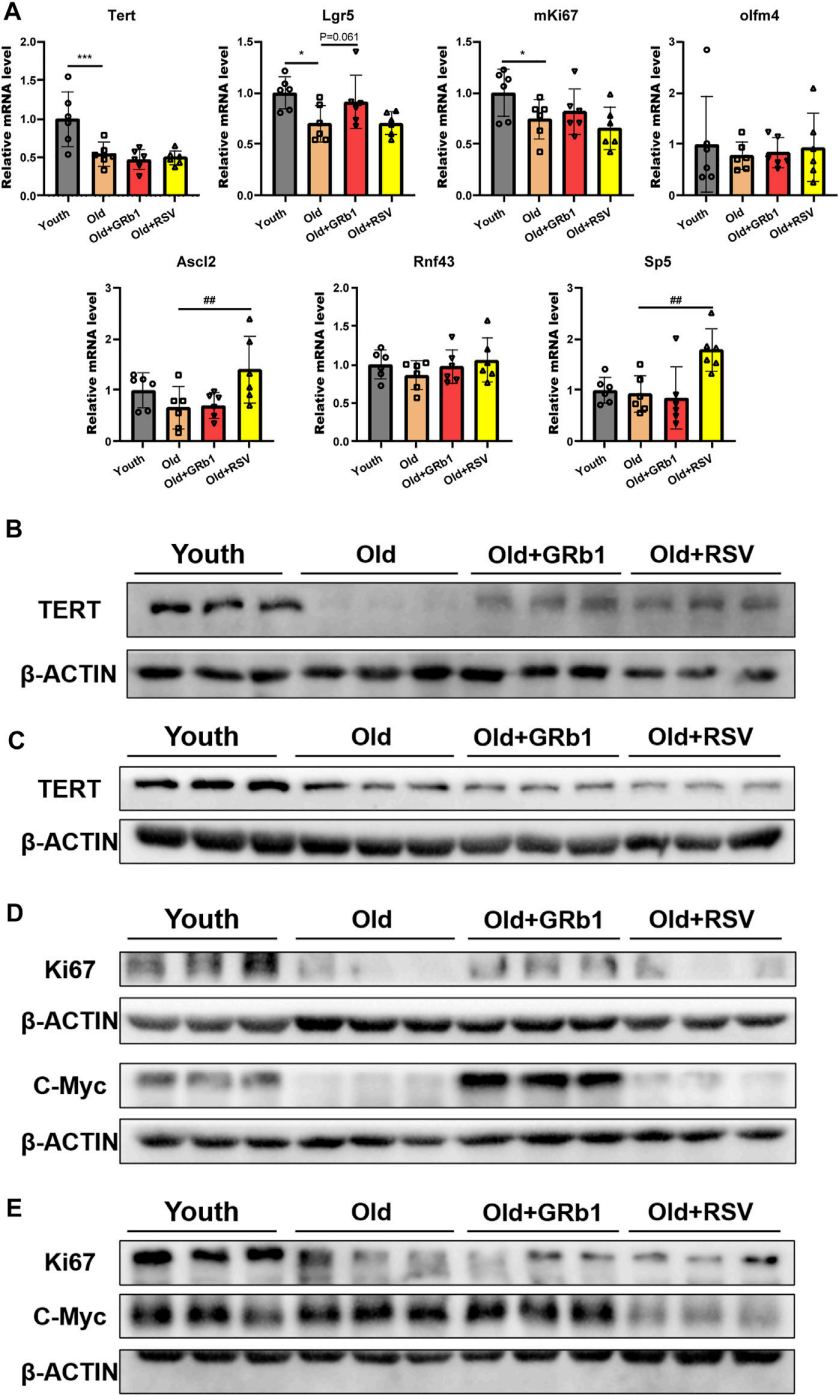
GRb1 was effective to improve the function of intestinal stem cells of old mice. **(A)** Relative mRNA expression levels of *Tert*, *Lgr5*, *mKi67*, *Olfm4*, *Ascl2*, *Rnf43*, and *Sp5* in the small intestines. **(B,C)** Images of western blot bands of TERT in the **(B)** jejunum and **(C)** colon. **(D,E)** Western blot results of Ki67 and c-Myc from the **(D)** jejunum and **(E)** colon. ^*^ *p* < 0.05, ^***^ *p* < 0.001, compared with the Youth group; ^##^ *p* < 0.01, compared with the Old group.

*Tert* was significantly reduced in the jejunum of old mice at both mRNA and protein levels compared to the Youth group (Figures 3A,B; Supplementary Figure 4A). The administration of GRb1 or RSV could not change the transcription of Tert in the jejunum of old mice (Figure 3A). However, both GRb1 and RSV could evidently increase the reduced TERT protein in the jejunum of old mice (Figure 3B; Supplementary Figure 4A). The protein level of TERT was also significantly lower in the colon of Old group than the Youth group, but GRb1 and RSV could not improve it (Figure 3C; Supplementary Figure 4B). The transcriptional level of *Lgr5* was significantly reduced in the jejunum of old mice compared to the young mice, and it was increased in the Old + GRb1 group although the increase was not significant (*p* = 0.061) (Figure 3A). RSV could not increase the mRNA level of *Lgr5* in the jejunum of old mice (Figure 3A). The transcriptional levels of other intestinal stem cell related genes, including *Olfm4*, *Ascl2, Rnf43*, and *Sp5*, showed no significant difference in the jejunum from Old and Youth groups (Figure 3A). However, RSV could increase *Ascl2* and *Sp5* in the jejunum of old mice (Figure 3A).

The proliferative ability of intestinal stem and progenitor cells was checked *via* testing the expression of mKi67 in the intestines of mice. Compared to the young mice, the mRNA level of *mKi67* was significantly reduced in the jejunum of the Old group, but GRb1 or RSV could not improve it (Figure 3A). The protein level of Ki67 was also significantly decreased in the jejunum of the Old group compared to the Youth group, and GRb1 could evidently increase it (Figure 3D; Supplementary Figure 4C). GRb1 increased the numbers of Ki67 positive cells in crypts of jejunum of the old mice (Supplementary Figure 5A). The administration of GRb1 also increased the reduced Ki67 protein in the colon of old mice, although the increase was not significant (*p* = 0.06) (Figure 3E; Supplementary Figures 4E, 5B). The protein level of c-Myc which is responsible for the transcription of pro-proliferative genes (Ruan et al., 2021) was significantly reduced in the jejunum of old mice, and GRb1 could significantly improve it (Figure 3D; Supplementary Figure 4D). The protein level of c-Myc was evidently higher in the colon of old mice than the Youth group, and it was decreased in the colon of Old + GRb1 and Old + RSV groups but not significantly (Figure 3E; Supplementary Figure 4F). These results indicated that GRb1 could improve the function of intestinal stem and progenitor cells.

### GRb1 regulated the expression of sirtuins in the intestines of old mice

**FIGURE 4:**
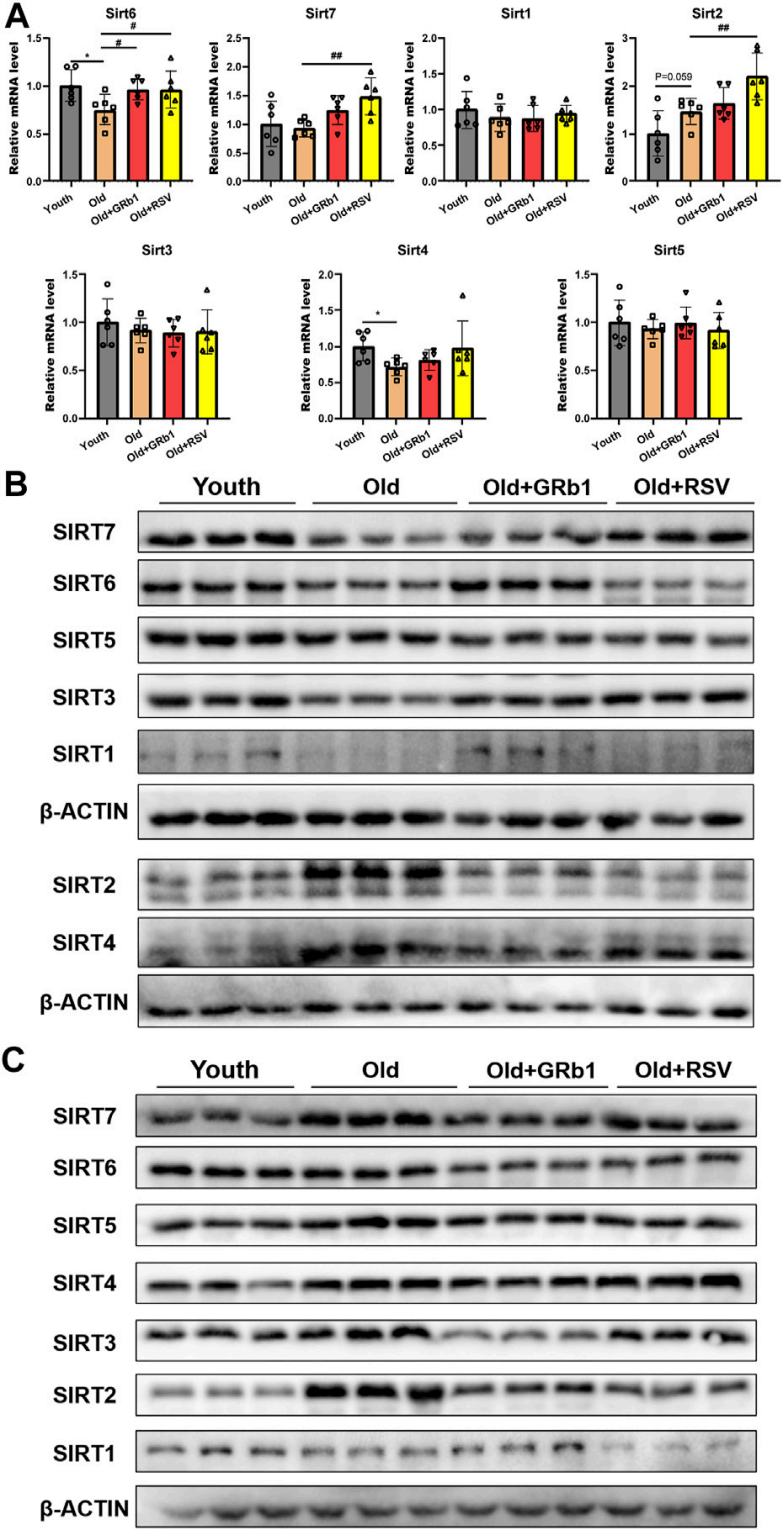
GRb1 improved the expression of Sirtuins in the intestines of old mice. **(A)** Relative mRNA expression levels of *Sirt 1-7* in the jejunum. **(B,C)** Images of Western blot bands of SIRT 1-7 in the (C) jejunum and (D) colon. ^*^ *p* < 0.05, compared with the Youth group; ^#^ *p* < 0.05, ^##^ *p* < 0.01, compared with the Old group.

The mRNA levels of *Sirt4* and *Sirt6* were all significantly decreased in the jejunum of old mice compared to the Youth group, and GRb1 or RSV could significantly increase the transcription of *Sirt6* but not *Sirt4* in old mice (Figure 4A). There was no significant difference of the transcriptional levels of *Sirt1*, *Sirt2*, *Sirt3*, *Sirt5*, and *Sirt7* between young and old mice (Figure 4A). However, the mRNA levels of *Sirt2* and *Sirt7* were all significantly increased in the jejunum of Old + RSV group compared to the Old group (Figure 4A). The protein levels of SIRT1, SIRT3, SIRT5, and SIRT6 were all lower in the jejunum of old mice than the Youth group, and GRb1 could rescue SIRT1 and SIRT6 in the jejunum of old mice (Figure 4B; Supplementary Figures 6A,C,E,F). The administration of GRb1 or RSV could also increase the expression of SIRT3 and SIRT7 in the jejunum of old mice, but the increase was not significant (Figure 4B; Supplementary Figures 6C,G). SIRT2 and SIRT4 were significantly increased in the jejunum of Old mice, but the protein level of SIRT4 was significantly reduced after administration of GRb1 or RSV (Figure 4B; Supplementary Figures 6B,D). However, the administration of GRb1 or RSV could not reduce the protein level of SIRT2 in the jejunum of old mice (Figure 4B; Supplementary Figure 6B).

The protein levels of SIRT1 and SIRT7 showed no significant difference among the Youth, Old, Old + GRb1 and Old + RSV groups (Figure 4C; Supplementary Figures 7A,G). The protein levels of SIRT2, SIRT3, SIRT4, SIRT5, and SIRT6 were all significantly increased in the colon of old mice compared to the Youth group, and administration of GRb1 or RSV could reduce SIRT2 in the colon of old mice (Figure 4C; Supplementary Figures 7B–F). These results indicated that GRb1 might improve the aging of intestines *via* regulating the expression sirtuins at both transcriptional and post-transcriptional levels.

### GRb1 changed the composition and function of gut microbiota of old mice

**FIGURE 5:**
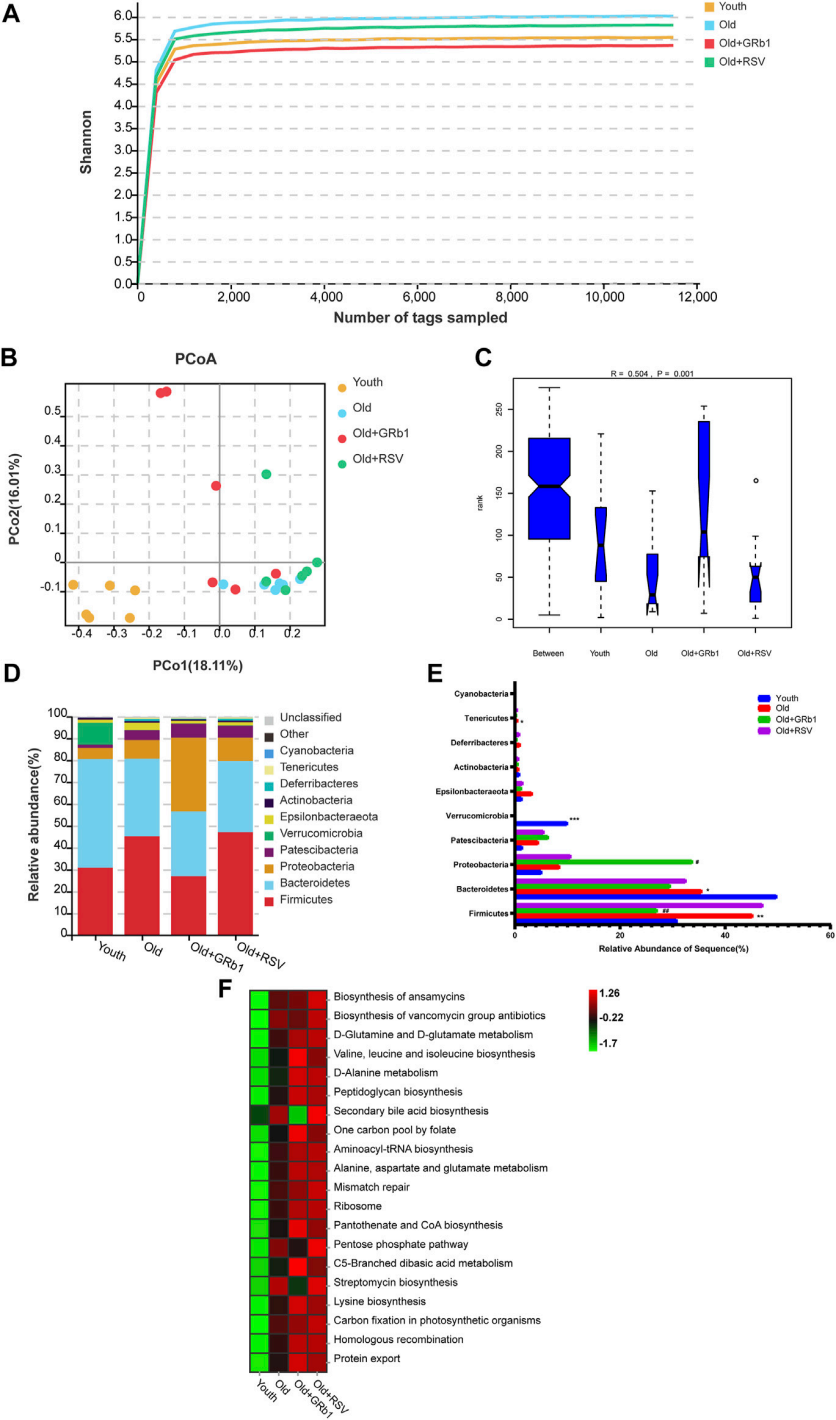
GRb1 changed the relative abundance and functions of gut microbiota of old mice. **(A)** Shannon rarefaction curves for each group. **(B)** The PCo analysis of the gut microbiota. **(C)** Analysis of similarity (ANOSIM) of the gut microbiota. **(D)** Relative abundance of the gut microbiota at phylum levels in mice. Different colors illustrated different flora. **(E)** Bar chart of proportional abundance of the gut microbiota at phylum levels in mice. **(F)** KEGG analysis showed the top 20 altered pathways of the gut microbiota. ^*^ *p* < 0.05, ^**^ *p* < 0.01, ^***^ *p* < 0.001, compared with the Youth group; ^#^ *p* < 0.05, ^##^ *p* < 0.01, compared with the Old group. PCo, Principle coordinates.

The 16S rRNA gene sequence was performed to analyze the composition and functions of the gut microbiota in mice (https://www.ncbi.nlm.nih.gov/sra/PRJNA856886). The Shannon rarefaction curves for every group had reached the saturated platform (Figure 5A), and the principle coordinates analysis (PCoA) showed that the Youth and the Old groups could be clearly distinguished (Figure 5B). Analysis of similarity (ANOSIM) showed that the rank of the Old group was lower than the Youth group, and the rank of the Old + GRb1 and Old + RSV groups was higher than the Old group (Figure 5C). At the phylum level, the abundance of *Firmicutes* and *Tenericutes* was significantly increased in the Old group compared to the Youth group, and the abundance of *Firmicutes* was significantly reduced after administration of GRb1 (Figures 5D,E). The abundance of *Bacteroidetes* and *Verrucomicrobia* was significantly reduced in the Old group compared to the Youth group (Figures 5D,E). The abundance of *Proteobacteria* was significantly increased in the Old + GRb1 group compared to the Old group (Figures 5D,E).

LEFse analysis showed there were 79 bacterial taxa differed in abundance between the Youth and Old groups, with 32 predominant for the Youth group and 47 predominant for the Old group (Supplementary Figures 8A,B). There were 57 bacterial taxa differed in abundance between the Old + GRb1 group and the Old group, with 21 predominant for the Old + GRb1 group and 36 predominant for the Old group (Supplementary Figures 8A,B). There were 24 bacterial taxa differed in abundance between the Old + RSV group and the Old group, with 12 predominant for the Old + RSV group and 12 predominant for the Old group (Supplementary Figures 10A,B). Compared to the Old group, there were three bacterial taxa predominant in all the three groups of the Youth, Old + GRb1 and Old + RSV, including Class *Actinobacteria*, Order *Corynebacteriales* and Family Corynebacteriaceae, and the Family Corynebacteriaceae belongs to the Order *Corynebacteriales*, the Order *Corynebacteriales* belongs to the Class *Actinobacteria* (Supplementary Figures 8A,B; Supplementary Figures 9A,B; Supplementary Figures 10A,B).

The top 20 altered pathways in the Kyoto Encyclopedia of Genes and Genomes (KEGG) pathway analysis were shown in Figure 5F. All the 20 pathways increased in the Old group, and GRb1 reduced four of them including “Biosynthesis of vancomycin group antibiotics,” “Pentose phosphate pathway,” “Streptomycin biosynthesis” and “Secondary bile acid biosynthesis,” although RSV could not reduce them. The other 16 pathways all increased after administration of GRb1 or RSV. These results indicated that the composition and functions of gut microbiota changed in old mice, and GRb1 might improve the intestinal aging partly through regulating the gut microbiota in old mice.

## Discussion

**FIGURE 6:**
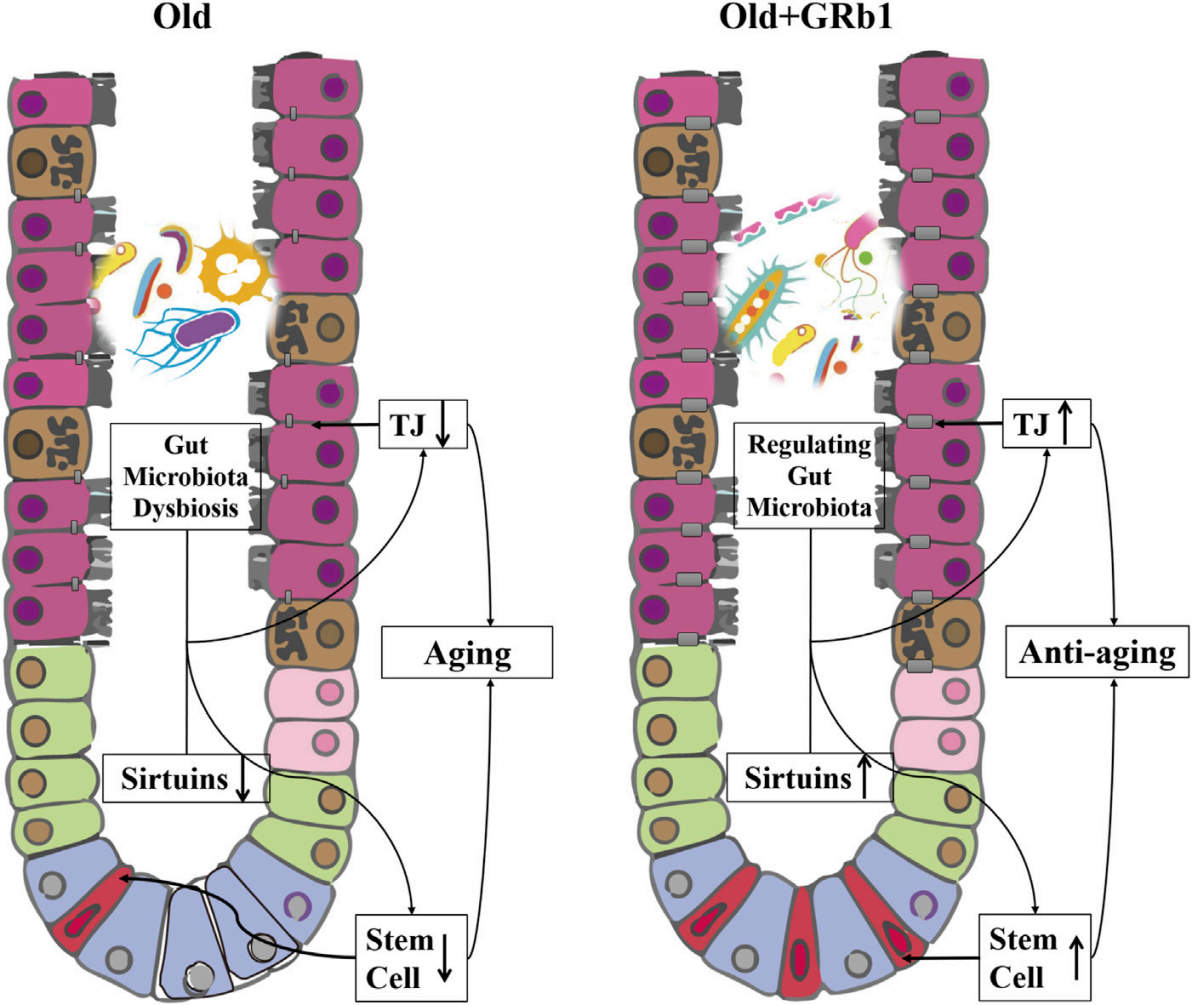
GRb1 improves the intestinal aging *via* up-regulating the expression sirtuins and modulating the gut microbiota. The downregulation of the members of sirtuins in the intestinal epithelium, especially in the small intestines, and the dysbiosis of the gut microbiota in the old mice are the two important mechanisms on inducing the aging of intestines. The integrity of the intestinal epithelium is affected because of the downregulation of tight junction components with the aging of intestines, and the stem and progenitor cells of the intestines is also reduced in the aged mice. GRb1 can upregulate the members of sirtuins family in the small intestines at transcriptional or post-transcriptional levels. At the same time, GRb1 can improve the dysbiosis of the gut microbiota in the old mice. Therefore, GRb1 might improve the aging of the intestinal epithelium via regulating the expression sirtuins and modulating the gut microbiota of the old mice.

We uncovered a new role of GRb1 on improving the intestinal aging of old mice (Figure 6). First, administration of GRb1 could increase the numbers of cells and reduce the accumulation of senescent cells in crypts of both small and large intestines from old mice. Then, GRb1 could improve the integrity of the intestinal epithelium *via* increasing the protein levels of the intestinal abundant Claudins in the intestinal epithelium of old mice. GRb1 could improve the function of intestinal stem and progenitor cells *via* upregulating the expression of Tert, Lgr5, mKi67, and c-Myc at transcriptional or posttranscriptional level in the small intestines of old mice. Then, it was demonstrated that GRb1 might improve intestinal aging through modulating the expression of members of Sirtuin family at both transcriptional and posttranscriptional levels in the intestines of old mice. Finally, 16S rDNA sequence results showed that GRb1 could modulate the composition and functions of gut microbiota in old mice, and it might be one of the mechanisms of GRb1 on improving intestinal aging of old mice.

Intestinal barrier defects are one of the hallmarks of intestinal aging (Arnold et al., 2021). It was reported that the serum LPS level is significantly higher in old mice than the young control mice (Shin et al., 2020), indicating the gut leaky of the old mice. In the present study, CLDN 2, 3, 7 and 15 all decreased in the small intestines of old mice and CLDN 15 also decreased in the large intestines of old mice. We speculated that the reduction of these intestinal abundance Claudins might be the important reason for the defects of the intestinal barrier of the old mice. Tight junction proteins, such as ZO-1, Occludin and CLDN 1, has also been found reduced in the ileum of aged rats (Ren et al., 2018). CLDN 2 and 15 have been reported to have important functions on regulating the paracellular flow of Na^+^ from the intestinal submucosa to dominate the absorption of glucose, amino acids and fats (Tamura et al., 2011; Wada et al., 2013). Therefore, the decrease of CLDN 2 and 15 in the small intestines of old mice might affect the absorption of nutrients. GRb1 might promote the nutrients absorption of aged mice *via* increasing the levels of CLDN 2 and 15 in the small intestines of them.

Previous study showed that GRb1 can promote the differentiation of muscle stem cells (Go et al., 2020). Neural stem cells in rats of Alzheimer’s disease models are also improved by GRb1 (Zhao et al., 2018). In the present study, GRb1 could improve the function of intestinal stem and progenitor cells *via* upregulating the expression of *Tert*, *Lgr5*, *mKi67*, and *c-Myc* in the small intestines of old mice. *Tert* has been confirmed to specifically express in the intestinal stem cells (Breault et al., 2008; Itzkovitz et al., 2011; Montgomery et al., 2011; Muñoz et al., 2012). Overexpression of TERT improves the fitness of intestinal barriers and produces a system delay in aging of mice (Tomás-Loba et al., 2008). GRb1 enhanced the protein level of TERT in the small intestines of old mice indicating its effects on anti-aging of intestinal stem cells. Ki67 has been used as the cell proliferation marker in both normal and cancer tissues (Chakritbudsabong et al., 2021; Silva et al., 2022). The increase of Ki67 in both small and large intestines of old mice after administration of GRb1 demonstrated that the number of proliferative cells increased in the intestinal crypts of them. We speculated the increase of the proliferative cells should be the direct mechanism on the increase of cells in crypts of intestines of the GRb1 treated mice.

Members of sirtuin family play the key role in aging and age-related disease (Kaitsuka et al., 2021). In the present study, GRb1 could increase the protein levels of SIRT1, SIRT3, SIRT6, and SIRT7 in the small intestines of old mice. SIRT1 becomes a target for the prevention and treatment of age-related cardiovascular and cerebrovascular diseases since it has been confirmed to have important function on preventing vascular aging (Begum et al., 2021). Recent study reported that LARP7 can ameliorate cellular senescence and aging through enhancing the activity of SIRT1 (Yan et al., 2021). The increase of the expression or activity of SIRT3 can extend the life span of human (Silaghi et al., 2021; Rose et al., 2003). Recently, it was found that reduced SIRT3 abundance in mice can exacerbate age-related periodontal disease (Chen et al., 2021). The level and activation of SIRT6 have been found to be reduced in the aging brain (Stein et al., 2021). The overexpression of SIRT6 can extend the life span of both mice and *Drosophila melanogaster* (Roichman et al., 2021; Taylor et al., 2022). SIRT7 has been found to antagonize stem cell aging *via* stabilizing heterochromatin (Sun and Dang, 2020; Bi et al., 2020). Therefore, the upregulation of SIRTs should be considered as one of the important mechanisms on improving the small intestinal aging of old mice.

In the present study, the composition and functions of gut microbiota changed in the old mice after administration of GRb1. At the phylum level of gut microbiota, the ratio of *Bacteroidetes*/*Firmicutes* decreased in the Old group compared to the Youth group, and administration of GRb1 could improve it. Many studies confirmed the decrease of the ratio of *Bacteroidetes*/*Firmicutes* in ob/ob mice compared with normal control mice (Turnbaugh et al., 2006; Abenavoli et al., 2019). The dysbiosis of the gut microbiota can increase the intestinal permeability (Zhang et al., 2010). Therefore, GRb1 might enhance the integrity of the intestinal epithelium *via* improving the dysbiosis of the gut microbiota in old mice. Compared to the Old group, the Class *Actinobacteria* was predominant in the Youth, Old + GRb1 and Old + RSV groups. *Actinobacteria* have been confirmed to be the biosynthetic factories which produce various bioactive metabolites, and many of these bioactive metabolites can be developed as drugs for human (Azman et al., 2019; Hussain et al., 2020; Jose et al., 2021). The pathways for “Valine, leucine and isoleucine biosynthesis” and “Lysine biosynthesis” significantly increased in old mice after administration with GRb1. Lysine, valine, leucine and isoleucine are essential amino acids for human, so the increase of the biosynthesis of them should be good for the health of the old mice. Hence, we speculated that the regulating of the gut microbiota might be another important mechanism of GRb1 on improving the intestinal aging of the old mice.

## Conclusion

In conclusion, GRb1 could improve the intestinal aging *via* regulating the expression of members of Sirtuin family in the intestinal epithelium at transcriptional or posttranscriptional levels and modulating the composition and functions of gut microbiota in the old mice (Figure 6).

## Data Availability

The datasets presented in this study can be found in online repositories. The names of the repository/repositories and accession number(s) can be found below: https://www.ncbi.nlm.nih.gov/sra/PRJNA856886.

## Abbreviations

ANOSIM, Analysis of similarity; Ascl2, achaete-scute family bHLH transcription factor 2; CLDN, Claudin; CMC-Na, CarboxyMethylCellulose-Na; EpCAM, epithelial cell adhesion molecule; ER, endoplasmic reticulum; H&E, hematoxylin and eosin; HRP, horseradish peroxidase; GRb1, Ginsenoside Rb1; KEGG, Kyoto Encyclopedia of Genes and Genomes; Lgr5, leucine rich repeat containing G protein-coupled receptor 5; mKi67, antigen identified by monoclonal antibody Ki 67; Myc, MYC proto-oncogene, bHLH transcription factor; OCT, optimal cutting temperature compound; Olfm4, olfactomedin 4; PCoA, principle coordinates analysis; qRT-PCR, quantitative real-time PCR; Rnf43, ring finger protein 43; RSV, resveratrol; Sirt, sirtuin; Sp5, Sp5 transcription factor; SPF, specific pathogen-free; Tert, telomerase reverse transcriptase; TGGR, total ginsenosides; TJ, tight junction; X-gal, beta-galactosidase.

## References

[B1] Abenavoli L. Scarpellini E. Colica C. Boccuto L. Salehi B. Sharifi-Rad J. (2019). Gut microbiota and obesity: A role for probiotics. Nutrients 11, E2690. 10.3390/nu11112690 31703257 PMC6893459

[B2] Arnold J. W. Roach J. Fabela S. Moorfield E. Ding S. Blue E. (2021). The pleiotropic effects of prebiotic galacto-oligosaccharides on the aging gut. Microbiome 9, 31. 10.1186/s40168-020-00980-0 33509277 PMC7845053

[B3] Azman A. S. Mawang C. I. Khairat J. E. Abubakar S. (2019). Actinobacteria-a promising natural source of anti-biofilm agents. Int. Microbiol. 22, 403–409. 10.1007/s10123-019-00066-4 30847714

[B4] Baghdadi M. B. Ayyaz A. Coquenlorge S. Chu B. Kumar S. Streutker C. (2022). Enteric glial cell heterogeneity regulates intestinal stem cell niches. Cell Stem Cell 29, 86–100.e6. 10.1016/j.stem.2021.10.004 34727519

[B5] Bai Y. Bao X. Mu Q. Fang X. Zhu R. Liu C. (2021). Ginsenoside Rb1, salvianolic acid B and their combination modulate gut microbiota and improve glucolipid metabolism in high-fat diet induced obese mice. PeerJ 9, e10598. 10.7717/peerj.10598 33604164 PMC7866888

[B6] Begum M. K. Konja D. Singh S. Chlopicki S. Wang Y. (2021). Endothelial SIRT1 as a target for the prevention of arterial aging: Promises and challenges. J. Cardiovasc. Pharmacol. 78, S63–s77. 10.1097/FJC.0000000000001154 34840264

[B7] Ben Othman S. Ido K. Masuda R. Gotoh S. Hosoda-Yabe R. Kitaguchi K. (2020). Senescence-accelerated mouse prone 8 mice exhibit specific morphological changes in the small intestine during senescence and after pectin supplemented diet. Exp. Gerontol. 142, 111099. 10.1016/j.exger.2020.111099 33011215

[B8] Bi S. Liu Z. Wu Z. Wang Z. Liu X. Wang S. (2020). SIRT7 antagonizes human stem cell aging as a heterochromatin stabilizer. Protein Cell 11, 483–504. 10.1007/s13238-020-00728-4 32504224 PMC7305295

[B9] Breault D. T. Min I. M. Carlone D. L. Farilla L. G. Ambruzs D. M. Henderson D. E. (2008). Generation of mTert-GFP mice as a model to identify and study tissue progenitor cells. Proc. Natl. Acad. Sci. U. S. A. 105, 10420–10425. 10.1073/pnas.0804800105 18650388 PMC2492454

[B10] Chakritbudsabong W. Sariya L. Jantahiran P. Chaisilp N. Chaiwattanarungruengpaisan S. Rungsiwiwut R. (2021). Generation of porcine induced neural stem cells using the sendai virus. Front. Vet. Sci. 8, 806785. 10.3389/fvets.2021.806785 35097051 PMC8790232

[B11] Chen J. Zhang Y. Gao J. Li T. Gan X. Yu H. (2021). Sirtuin 3 deficiency exacerbates age-related periodontal disease. J. Periodontal Res. 56, 1163–1173. 10.1111/jre.12930 34591326 PMC9293453

[B12] Chen S. Li X. Wang Y. Mu P. Chen C. Huang P. (2019). Ginsenoside Rb1 attenuates intestinal ischemia/reperfusion-induced inflammation and oxidative stress via activation of the PI3K/Akt/Nrf2 signaling pathway. Mol. Med. Rep. 19, 3633–3641. 10.3892/mmr.2019.10018 30864725 PMC6471656

[B13] Cheng Y. Shen L. H. Zhang J. T. (2005). Anti-amnestic and anti-aging effects of ginsenoside Rg1 and Rb1 and its mechanism of action. Acta Pharmacol. Sin. 26, 143–149. 10.1111/j.1745-7254.2005.00034.x 15663889

[B14] Dong J. Y. Xia K. J. Liang W. Liu L. L. Yang F. Fang X. S. (2021). Ginsenoside Rb1 alleviates colitis in mice via activation of endoplasmic reticulum-resident E3 ubiquitin ligase Hrd1 signaling pathway. Acta Pharmacol. Sin. 42, 1461–1471. 10.1038/s41401-020-00561-9 33268823 PMC8379258

[B15] Friedrich A. W. (2019). Control of hospital acquired infections and antimicrobial resistance in europe: The way to go. Wien. Med. Wochenschr. 169, 25–30. 10.1007/s10354-018-0676-5 PMC6373234 30623278

[B16] Gámez-García A. Vazquez B. N. (2021). Nuclear sirtuins and the aging of the immune system. Genes (Basel) 12, 1856. 10.3390/genes12121856 34946805 PMC8701065

[B17] Go G. Y. Jo A. Seo D. W. Kim W. Y. Kim Y. K. So E. Y. (2020). Ginsenoside Rb1 and Rb2 upregulate Akt/mTOR signaling-mediated muscular hypertrophy and myoblast differentiation. J. Ginseng Res. 44, 435–441. 10.1016/j.jgr.2019.01.007 32372865 PMC7195574

[B18] Hamidian M. Foroughinia F. Haghighat S. Attar A. Haem E. (2022). Protective effects of Panax ginseng against doxorubicin-induced cardiac toxicity in patients with non-metastatic breast cancer: A randomized, double-blind, placebo-controlled clinical trial. J. Oncol. Pharm. Pract. 2022, 107815522211185. 10.1177/10781552221118530 35975564

[B19] Hassan Z. A. Zauszkiewicz-Pawlak A. Abdelrahman S. A. Algaidi S. Desouky M. Shalaby S. M. (2017). Morphological alterations in the jejunal mucosa of aged rats and the possible protective role of green tea. Folia histochem. Cytobiol. 55, 124–139. 10.5603/FHC.a2017.0012 28813122

[B20] He J. Y. Hong Q. Chen B. X. Cui S. Y. Liu R. Cai G. Y. (2022). Ginsenoside Rb1 alleviates diabetic kidney podocyte injury by inhibiting aldose reductase activity. Acta Pharmacol. Sin. 43, 342–353. 10.1038/s41401-021-00788-0 34811512 PMC8791932

[B21] Hoffmeyer K. Raggioli A. Rudloff S. Anton R. Hierholzer A. Del Valle I. (2012). Wnt/β-catenin signaling regulates telomerase in stem cells and cancer cells. Science 336, 1549–1554. 10.1126/science.1218370 22723415

[B22] Hussain A. Hassan Q. P. Shouche Y. S. (2020). New approaches for antituberculosis leads from Actinobacteria. Drug Discov. Today 25, 2335–2342. 10.1016/j.drudis.2020.10.005 33069935

[B23] Hyun S. H. Bhilare K. D. Park C. K. Kim J. H. (2022). Effects of Panax ginseng and ginsenosides on oxidative stress and cardiovascular diseases: Pharmacological and therapeutic roles. J. Ginseng Res. 46, 33–38. 10.1016/j.jgr.2021.07.007 35058725 PMC8753520

[B24] Ishaq M. Khan A. Bacha A. S. Shah T. Hanif A. Ahmad A. A. (2021). Microbiota targeted interventions of probiotic lactobacillus as an anti-ageing approach: A review. Antioxidants (Basel) 10, 1930. 10.3390/antiox10121930 34943033 PMC8750034

[B25] Itzkovitz S. Lyubimova A. Blat I. C. Maynard M. Van Es J. Lees J. (2011). Single-molecule transcript counting of stem-cell markers in the mouse intestine. Nat. Cell Biol. 14, 106–114. 10.1038/ncb2384 22119784 PMC3292866

[B26] Jose P. A. Maharshi A. Jha B. (2021). Actinobacteria in natural products research: Progress and prospects. Microbiol. Res. 246, 126708. 10.1016/j.micres.2021.126708 33529791

[B27] Kaitsuka T. Matsushita M. Matsushita N. (2021). Regulation of hypoxic signaling and oxidative stress via the MicroRNA-SIRT2 Axis and its relationship with aging-related diseases. Cells 10, 3316. 10.3390/cells10123316 34943825 PMC8699081

[B28] Lei Z. Maeda T. Tamura A. Nakamura T. Yamazaki Y. Shiratori H. (2012). EpCAM contributes to formation of functional tight junction in the intestinal epithelium by recruiting claudin proteins. Dev. Biol. 371, 136–145. 10.1016/j.ydbio.2012.07.005 22819673

[B29] Lei Z. Wu H. Yang Y. Hu Q. Lei Y. Liu W. (2021a). Ovariectomy impaired hepatic glucose and lipid homeostasis and altered the gut microbiota in mice with different diets. Front. Endocrinol. 12, 708838. 10.3389/fendo.2021.708838 PMC8278766 34276568

[B30] Lei Z. Yang L. Lei Y. Yang Y. Zhang X. Song Q. (2021b). High dose lithium chloride causes colitis through activating F4/80 positive macrophages and inhibiting expression of Pigr and Claudin-15 in the colon of mice. Toxicology 457, 152799. 10.1016/j.tox.2021.152799 33901603

[B31] Lei Z. Yang Y. Liu S. Lei Y. Yang L. Zhang X. (2020). Dihydroartemisinin ameliorates dextran sulfate sodium induced inflammatory bowel diseases in mice. Bioorg. Chem. 100, 103915. 10.1016/j.bioorg.2020.103915 32450383

[B32] Li X. Khan I. Xia W. Huang G. Liu L. Law B. Y. K. (2021). Icariin enhances youth-like features by attenuating the declined gut microbiota in the aged mice. Pharmacol. Res. 168, 105587. 10.1016/j.phrs.2021.105587 33798737

[B33] Lima-Fontes M. Meira L. Barata P. Falcão M. Carneiro Â. (2021). Gut microbiota and age-related macular degeneration: A growing partnership. Surv. Ophthalmol. 67, 883. 10.1016/j.survophthal.2021.11.009 34843745

[B34] Lin Z. Xie R. Zhong C. Huang J. Shi P. Yao H. (2022). Recent progress (2015-2020) in the investigation of the pharmacological effects and mechanisms of ginsenoside Rb(1), a main active ingredient in Panax ginseng Meyer. J. Ginseng Res. 46, 39–53. 10.1016/j.jgr.2021.07.008 35058726 PMC8753521

[B35] López-Otín C. Blasco M. A. Partridge L. Serrano M. Kroemer G. (2013). The hallmarks of aging. Cell 153, 1194–1217. 10.1016/j.cell.2013.05.039 23746838 PMC3836174

[B36] Lukiw W. J. Arceneaux L. Li W. Bond T. Zhao Y. (2021). Gastrointestinal (GI)-Tract microbiome derived neurotoxins and their potential contribution to inflammatory neurodegeneration in Alzheimer's disease (AD). J. Alzheimers Dis. Park. 11, 525. 10.4172/2161-0460.1000525 PMC8395586 34457996

[B37] Montgomery R. K. Carlone D. L. Richmond C. A. Farilla L. Kranendonk M. E. Henderson D. E. (2011). Mouse telomerase reverse transcriptase (mTert) expression marks slowly cycling intestinal stem cells. Proc. Natl. Acad. Sci. U. S. A. 108, 179–184. 10.1073/pnas.1013004108 21173232 PMC3017192

[B38] Muñoz J. Stange D. E. Schepers A. G. Van De Wetering M. Koo B. K. Itzkovitz S. (2012). The Lgr5 intestinal stem cell signature: Robust expression of proposed quiescent '+4' cell markers. Embo J. 31, 3079–3091. 10.1038/emboj.2012.166 22692129 PMC3400017

[B39] Niu K. M. Bao T. Gao L. Ru M. Li Y. Jiang L. (2021). The impacts of short-term NMN supplementation on serum metabolism, fecal microbiota, and telomere length in pre-aging phase. Front. Nutr. 8, 756243. 10.3389/fnut.2021.756243 34912838 PMC8667784

[B40] Parrish A. R. (2017). The impact of aging on epithelial barriers. Tissue Barriers 5, e1343172. 10.1080/21688370.2017.1343172 28686506 PMC5788442

[B41] Pénzes L. (1984). Intestinal response in aging: Changes in reserve capacity. Acta Med. hung. 41, 263–277. 6393034

[B42] Qin G. W. Lu P. Peng L. Jiang W. (2021). Ginsenoside Rb1 inhibits cardiomyocyte autophagy via PI3K/Akt/mTOR signaling pathway and reduces myocardial ischemia/reperfusion injury. Am. J. Chin. Med. 49, 1913–1927. 10.1142/S0192415X21500907 34775933

[B43] Ren M. Li H. Fu Z. Li Q. (2021). Succession analysis of gut microbiota structure of participants from long-lived families in hechi, guangxi, China. Microorganisms 9, 2524. 10.3390/microorganisms9122524 34946126 PMC8703768

[B44] RenW.WuJ.LiL.LuY.ShaoY.QiY. (2018). Glucagon-Like peptide-2 improve intestinal mucosal barrier function in aged rats. J. Nutr. Health Aging 22, 731–738. 10.1007/s12603-018-1022-8 29806863

[B45] Roichman A. Elhanati S. Aon M. A. Abramovich I. Di Francesco A. Shahar Y. (2021). Restoration of energy homeostasis by SIRT6 extends healthy lifespan. Nat. Commun. 12, 3208. 10.1038/s41467-021-23545-7 34050173 PMC8163764

[B46] Rose G. Dato S. Altomare K. Bellizzi D. Garasto S. Greco V. (2003). Variability of the SIRT3 gene, human silent information regulator Sir2 homologue, and survivorship in the elderly. Exp. Gerontol. 38, 1065–1070. 10.1016/s0531-5565(03)00209-2 14580859

[B47] Ruan Y. Kim H. N. Ogana H. A. Wan Z. Hurwitz S. Nichols C. (2021). Preclinical evaluation of a novel dual targeting PI3kδ/BRD4 inhibitor, SF2535, in B-cell acute lymphoblastic leukemia. Front. Oncol. 11, 766888. 10.3389/fonc.2021.766888 34926269 PMC8671162

[B48] Ruiz-Gonzalez C. Cardona D. Rodriguez-Arrastia M. Ropero-Padilla C. Rueda-Ruzafa L. Carvajal F. (2022). Effects of probiotics on cognitive and emotional functions in healthy older adults: Protocol for a double-blind randomized placebo-controlled crossover trial. Res. Nurs. Health 45, 274–286. 10.1002/nur.22209 35080033

[B49] Sharma R. (2022). Emerging interrelationship between the gut microbiome and cellular senescence in the context of aging and disease: Perspectives and therapeutic opportunities. Probiotics Antimicrob. Proteins 14, 648–663. 10.1007/s12602-021-09903-3 34985682 PMC8728710

[B50] Shin H. E. Kwak S. E. Zhang D. D. Lee J. Yoon K. J. Cho H. S. (2020). Effects of treadmill exercise on the regulation of tight junction proteins in aged mice. Exp. Gerontol. 141, 111077. 10.1016/j.exger.2020.111077 32898618

[B51] Silaghi C. N. Farcaș M. Crăciun A. M. (2021). Sirtuin 3 (SIRT3) pathways in age-related cardiovascular and neurodegenerative diseases. Biomedicines 9, 1574. 10.3390/biomedicines9111574 34829803 PMC8615405

[B52] Silva L. C. Faustino I. S. P. Cantadori G. R. Santos-Silva A. R. Vargas P. A. Lopes M. A. (2022). Adenocarcinoma not otherwise specified (NOS) arising in the sublingual gland: Rare case report and follow-up. Oral Oncol. 126, 105754. 10.1016/j.oraloncology.2022.105754 35123257

[B53] Stein D. Mizrahi A. Golova A. Saretzky A. Venzor A. G. Slobodnik Z. (2021). Aging and pathological aging signatures of the brain: Through the focusing lens of SIRT6. Aging (Albany NY) 13, 6420–6441. 10.18632/aging.202755 33690173 PMC7993737

[B54] Sun L. Dang W. (2020). SIRT7 slows down stem cell aging by preserving heterochromatin: A perspective on the new discovery. Protein Cell 11, 469–471. 10.1007/s13238-020-00735-5 32435977 PMC7305289

[B55] Tamura A. Hayashi H. Imasato M. Yamazaki Y. Hagiwara A. Wada M. (2011). Loss of claudin-15, but not claudin-2, causes Na+ deficiency and glucose malabsorption in mouse small intestine. Gastroenterology 140, 913–923. 10.1053/j.gastro.2010.08.006 20727355

[B56] Taylor J. R. Wood J. G. Mizerak E. Hinthorn S. Liu J. Finn M. (2022). Sirt6 regulates lifespan in *Drosophila melanogaster* . Proc. Natl. Acad. Sci. U. S. A. 119, e2111176119. 10.1073/pnas.2111176119 35091469 PMC8812521

[B57] Tomás-Loba A. Flores I. Fernández-Marcos P. J. Cayuela M. L. Maraver A. Tejera A. (2008). Telomerase reverse transcriptase delays aging in cancer-resistant mice. Cell 135, 609–622. 10.1016/j.cell.2008.09.034 19013273

[B58] Toyokawa Y. Takagi T. Uchiyama K. Mizushima K. Inoue K. Ushiroda C. (2019). Ginsenoside Rb1 promotes intestinal epithelial wound healing through extracellular signal-regulated kinase and Rho signaling. J. Gastroenterol. Hepatol. 34, 1193–1200. 10.1111/jgh.14532 30394577

[B59] Tran L. Greenwood-Van Meerveld B. (2013). Age-associated remodeling of the intestinal epithelial barrier. J. Gerontol. A Biol. Sci. Med. Sci. 68, 1045–1056. 10.1093/gerona/glt106 23873964 PMC3738030

[B60] Turnbaugh P. J. Ley R. E. Mahowald M. A. Magrini V. Mardis E. R. Gordon J. I. (2006). An obesity-associated gut microbiome with increased capacity for energy harvest. Nature 444, 1027–1031. 10.1038/nature05414 17183312

[B61] Wada M. Tamura A. Takahashi N. Tsukita S. (2013). Loss of claudins 2 and 15 from mice causes defects in paracellular Na+ flow and nutrient transport in gut and leads to death from malnutrition. Gastroenterology 144, 369–380. 10.1053/j.gastro.2012.10.035 23089202

[B62] Watroba M. Szukiewicz D. (2021). Sirtuins at the service of healthy longevity. Front. Physiol. 12, 724506. 10.3389/fphys.2021.724506 34899370 PMC8656451

[B63] Wu C. J. Mannan P. Lu M. Udey M. C. (2013). Epithelial cell adhesion molecule (EpCAM) regulates claudin dynamics and tight junctions. J. Biol. Chem. 288, 12253–12268. 10.1074/jbc.M113.457499 23486470 PMC3636909

[B64] Xiong Y. Shen L. Liu K. J. Tso P. Xiong Y. Wang G. (2010). Antiobesity and antihyperglycemic effects of ginsenoside Rb1 in rats. Diabetes 59, 2505–2512. 10.2337/db10-0315 20682695 PMC3279544

[B65] Yan P. Li Z. Xiong J. Geng Z. Wei W. Zhang Y. (2021). LARP7 ameliorates cellular senescence and aging by allosterically enhancing SIRT1 deacetylase activity. Cell Rep. 37, 110038. 10.1016/j.celrep.2021.110038 34818543

[B66] Yang C. Wang W. Deng P. Li C. Zhao L. Gao H. (2021a). Fibroblast growth factor 21 modulates microglial polarization that attenuates neurodegeneration in mice and cellular models of Parkinson's disease. Front. Aging Neurosci. 13, 778527. 10.3389/fnagi.2021.778527 35002679 PMC8727910

[B67] Yang X. Dong B. An L. Zhang Q. Chen Y. Wang H. (2021b). Ginsenoside Rb1 ameliorates glycemic disorder in mice with high fat diet-induced obesity via regulating gut microbiota and amino acid metabolism. Front. Pharmacol. 12, 756491. 10.3389/fphar.2021.756491 34899310 PMC8654325

[B68] Yao H. Wan J. Y. Zeng J. Huang W. H. Sava-Segal C. Li L. (2018). Effects of compound K, an enteric microbiome metabolite of ginseng, in the treatment of inflammation associated colon cancer. Oncol. Lett. 15, 8339–8348. 10.3892/ol.2018.8414 29805567 PMC5950138

[B69] Zhang W. Gu Y. Chen Y. Deng H. Chen L. Chen S. (2010). Intestinal flora imbalance results in altered bacterial translocation and liver function in rats with experimental cirrhosis. Eur. J. Gastroenterol. Hepatol. 22, 1481–1486. 10.1097/MEG.0b013e32833eb8b0 20739895

[B70] Zhang Y. Zhang S. Li B. Luo Y. Gong Y. Jin X. (2021). Gut microbiota dysbiosis promotes age-related atrial fibrillation by lipopolysaccharide and glucose-induced activation of NLRP3-inflammasome. Cardiovasc. Res. 118, 785–797. 10.1093/cvr/cvab114 33757127

[B71] Zhao A. Liu N. Yao M. Zhang Y. Yao Z. Feng Y. (2022a). A review of neuroprotective effects and mechanisms of ginsenosides from Panax ginseng in treating ischemic stroke. Front. Pharmacol. 13, 946752. 10.3389/fphar.2022.946752 35873557 PMC9302711

[B72] Zhao J. Lu S. Yu H. Duan S. Zhao J. (2018). Baicalin and ginsenoside Rb1 promote the proliferation and differentiation of neural stem cells in Alzheimer's disease model rats. Brain Res. 1678, 187–194. 10.1016/j.brainres.2017.10.003 29038007

[B73] Zhao Q. Liu Y. Zhang S. Zhao Y. Wang C. Li K. (2022b). Studies on the regulation and molecular mechanism of Panax ginseng saponins on senescence and related behaviors of *Drosophila melanogaster* . Front. Aging Neurosci. 14, 870326. 10.3389/fnagi.2022.870326 35795238 PMC9252430

[B74] Zhao Y. Jaber V. Lukiw W. J. (2021). Gastrointestinal tract microbiome-derived pro-inflammatory neurotoxins in Alzheimer's disease. J. Aging Sci. 9, 002. 10.35248/2329-8847.21.s5.002 34671696 PMC8525708

[B75] Zhou F. Zhang P. Chen X. Yan J. Yao J. Yu Z. (2016). Ginsenoside Rb1 protects the intestinal mucosal barrier following peritoneal air exposure. Exp. Ther. Med. 12, 2563–2567. 10.3892/etm.2016.3639 27703510 PMC5038908

[B76] Zhou P. Xie W. He S. Sun Y. Meng X. Sun G. (2019). Ginsenoside Rb1 as an anti-diabetic agent and its underlying mechanism analysis. Cells 8, E204. 10.3390/cells8030204 30823412 PMC6468558

