# Supplementary material for: Ginsenoside Rb1 improves intestinal aging via regulating the expression of sirtuins in the intestinal epithelium and modulating the gut microbiota of mice
Source: Front Pharmacol. 2022 Sep 27;13:991597. doi: 10.3389/fphar.2022.991597 (PMC9552198; doi:10.3389/fphar.2022.991597)
Supplement: Supplementary file 1 [file DataSheet1.docx]

Supplementary Material


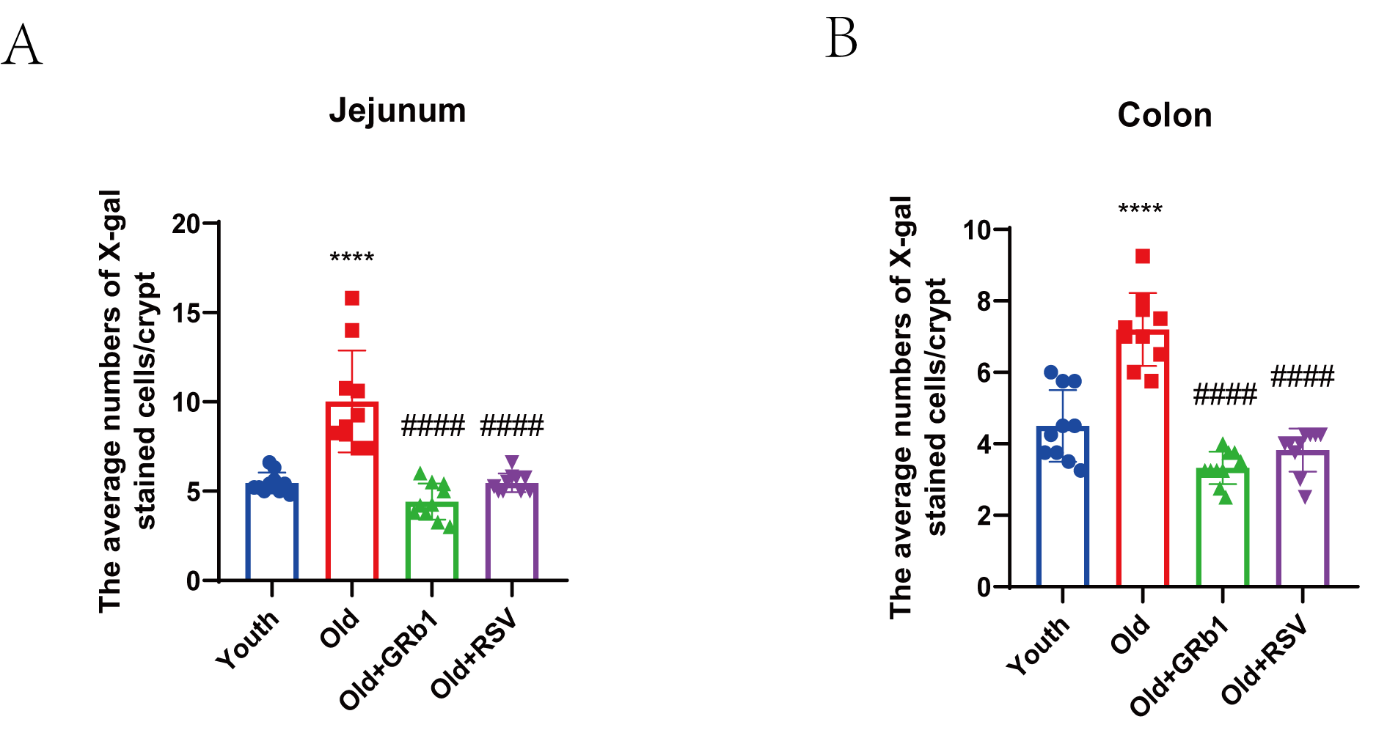


**Supplementary Figure 1. Quantitative Analysis of the X-gal staining Results in Figure 1**

**A-B**. Quantification data of the X-gal staining results of (A) Fig 1F and (B) Fig. 1G, respectively. The average numbers of the X-gal stained cells in the crypts of each image were calculated, and 10 images were counted of each group. ***P<0.001 compared with Youth group; ###P<0.001 compared with Old group.


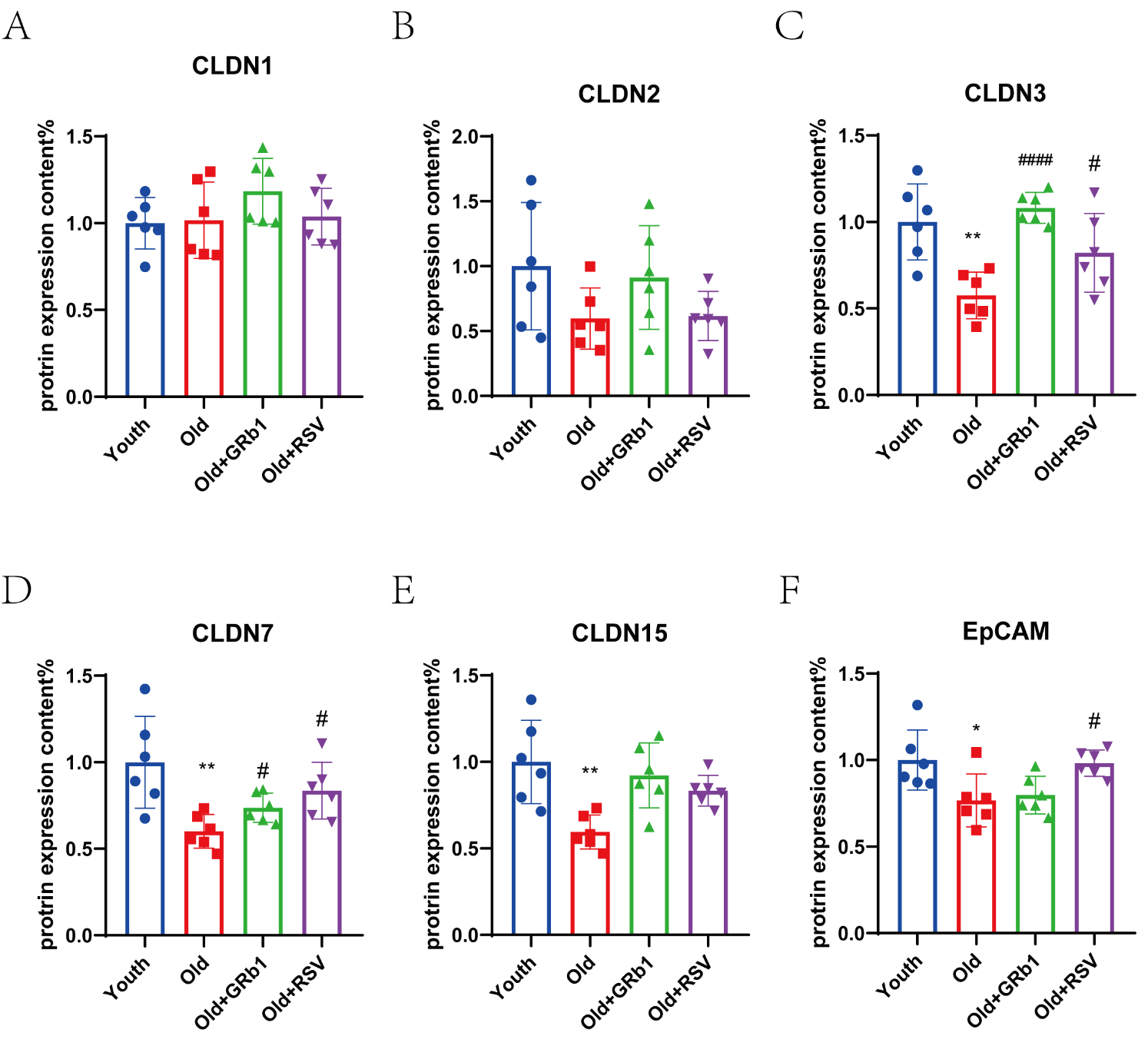


**Supplementary Figure 2. Quantitative Analysis of the Western Blot Results in Figure 2A**

**A-F**. Quantification data of the western blot results of (A) CLDN 1, (B) CLDN 2, (C) CLDN 3, (D) CLDN 7, (E) CLDN 15 and (F) EpCAM, respectively. 6 mice in each group for 2 times independent experiments. *P<0.05, **P<0.01 compared with Youth group; #P<0.05, ###P<0.001 compared with Old group.


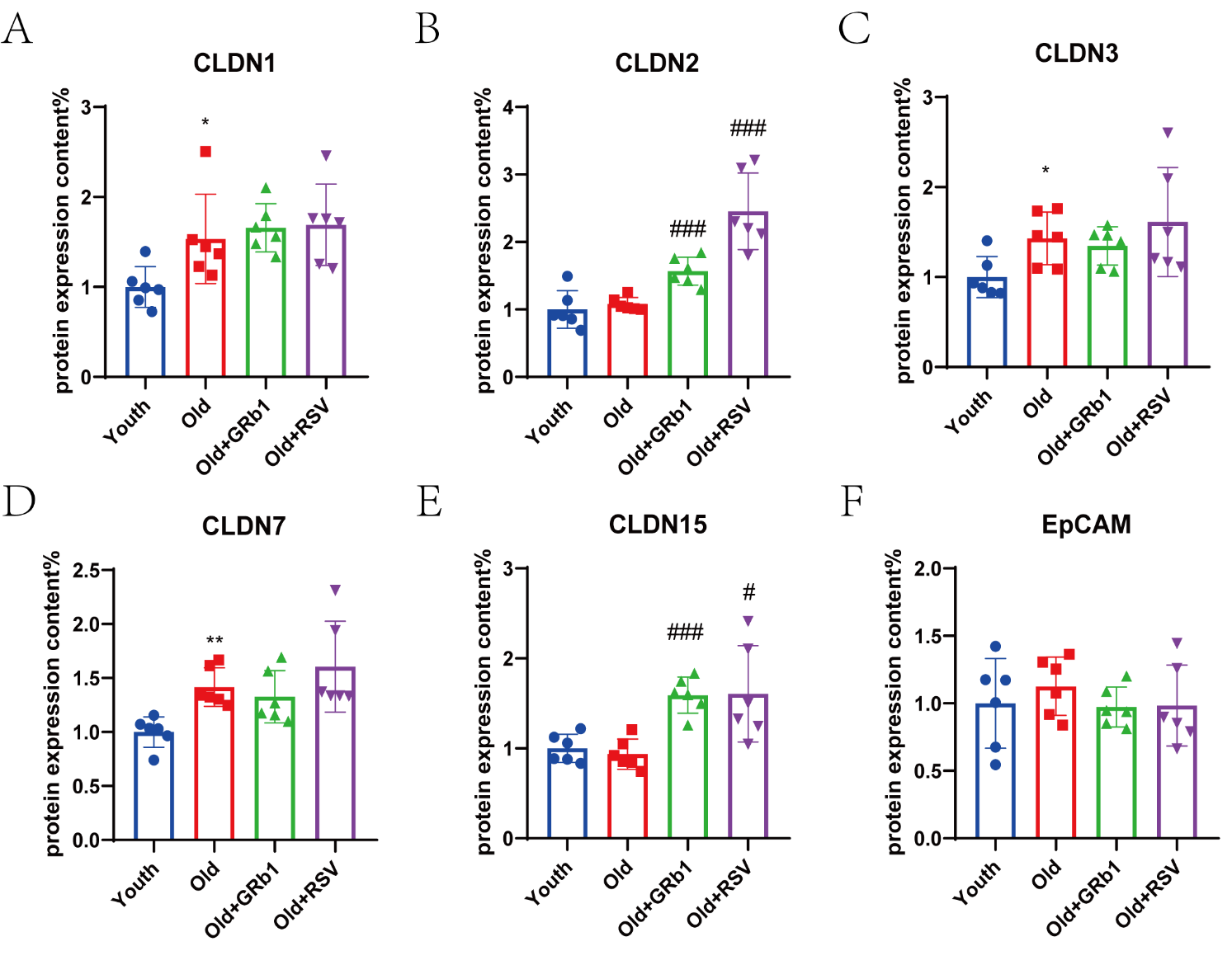


**Supplementary Figure 3. Quantitative Analysis of the Western Blot Results in Figure 2B**

**A-F**. Quantification data of the western blot results of (A) CLDN 1, (B) CLDN 2, (C) CLDN 3, (D) CLDN 7, (E) CLDN 15 and (F) EpCAM, respectively. 6 mice in each group for 2 times independent experiments. *P<0.05, **P<0.01 compared with Youth group; #P<0.05, ###P<0.001 compared with Old group.


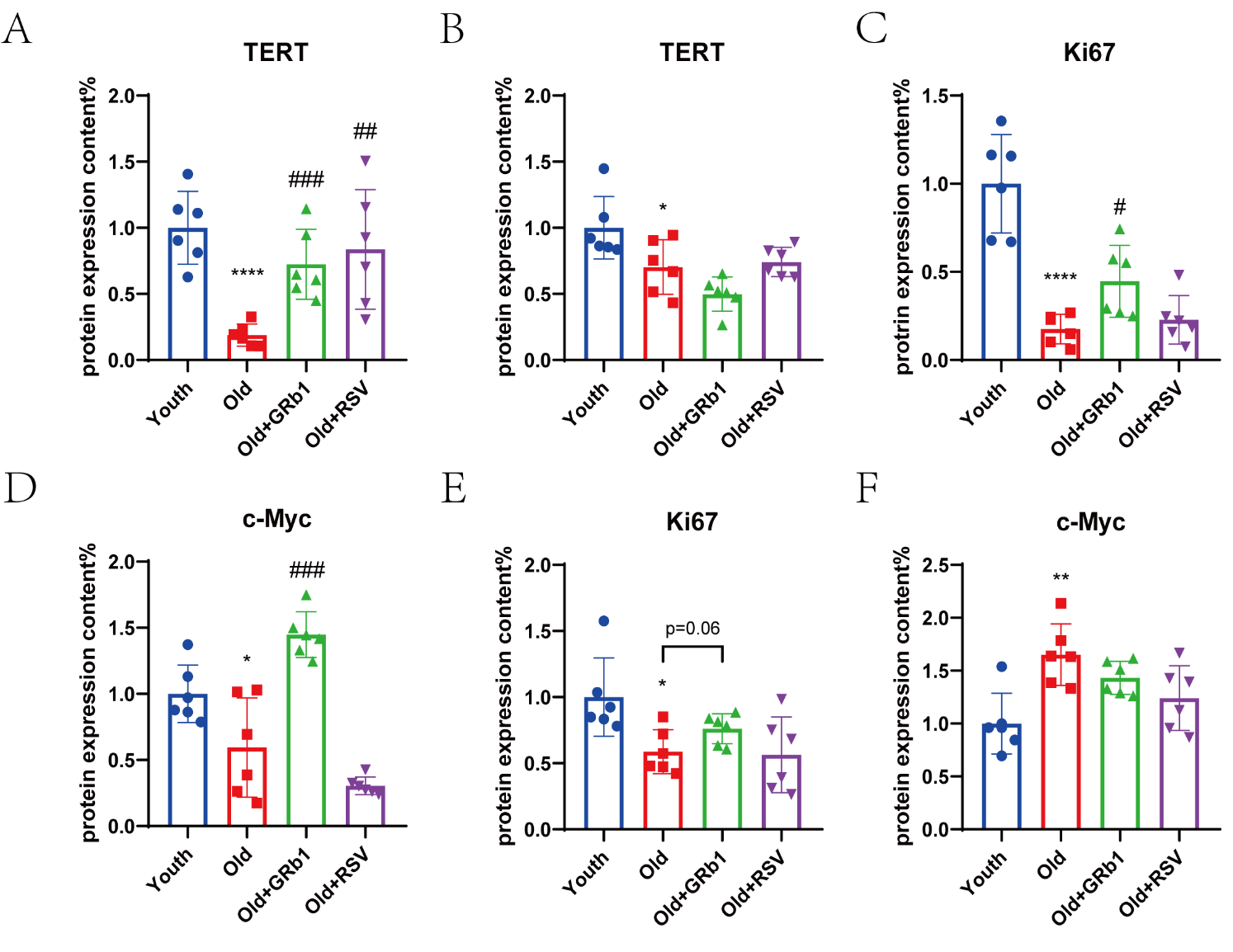


**Supplementary Figure 4. Quantitative Analysis of the Western Blot Results in Figure 3**

**A-B**. Quantification data of the western blot results of (A) Fig. 3B and (B) Fig. 3C, respectively. **C-D**. Quantification data of the western blot results of (C) Ki67 in Fig. 3D and (D) c-Myc in Fig. 3D, respectively. **E-F**. Quantification data of the western blot results of (E) Ki67 in Fig. 3E and (F) c-Myc in Fig. 3E, respectively. 6 mice in each group for 2 times independent experiments. *P<0.05, **P<0.01, ***P<0.001 compared with Youth group; #P<0.05, ##P<0.01, ###P<0.001 compared with Old group.


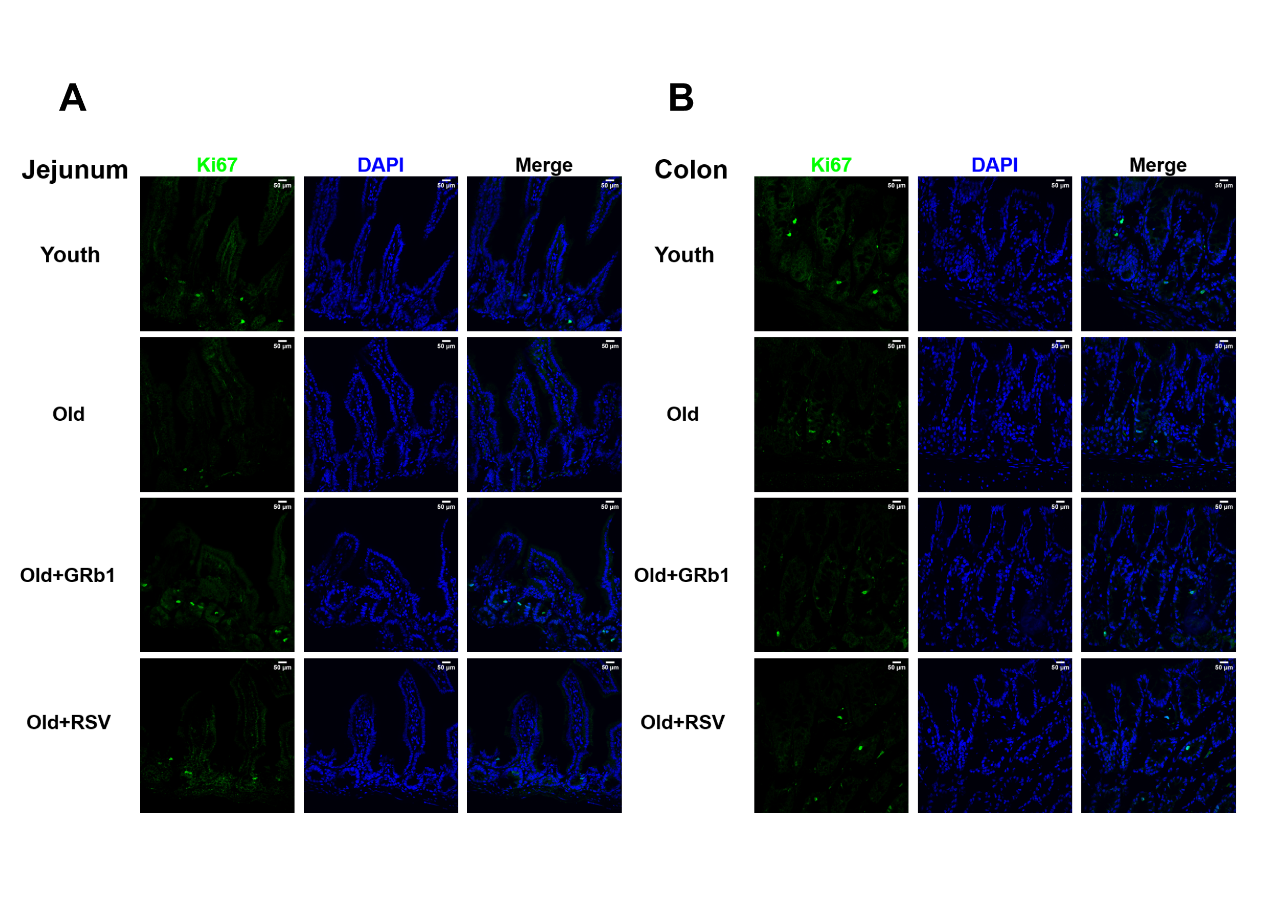


**Supplementary Figure 5.** **Ginsenoside Rb1 was effective to improve the expression of mKi67 in the intestinal epithelium of old mice**

**A-B**. Representative images of immunofluorescence staining with antibodies to Ki67 of frozen sections of (A) jejunum and (B) colon of mice from Youth, Old, Old+GRb1 and Old+RSV groups. Scale bar, 50 μm. GRb1, Ginsenoside Rb1; RSV, Resveratrol.


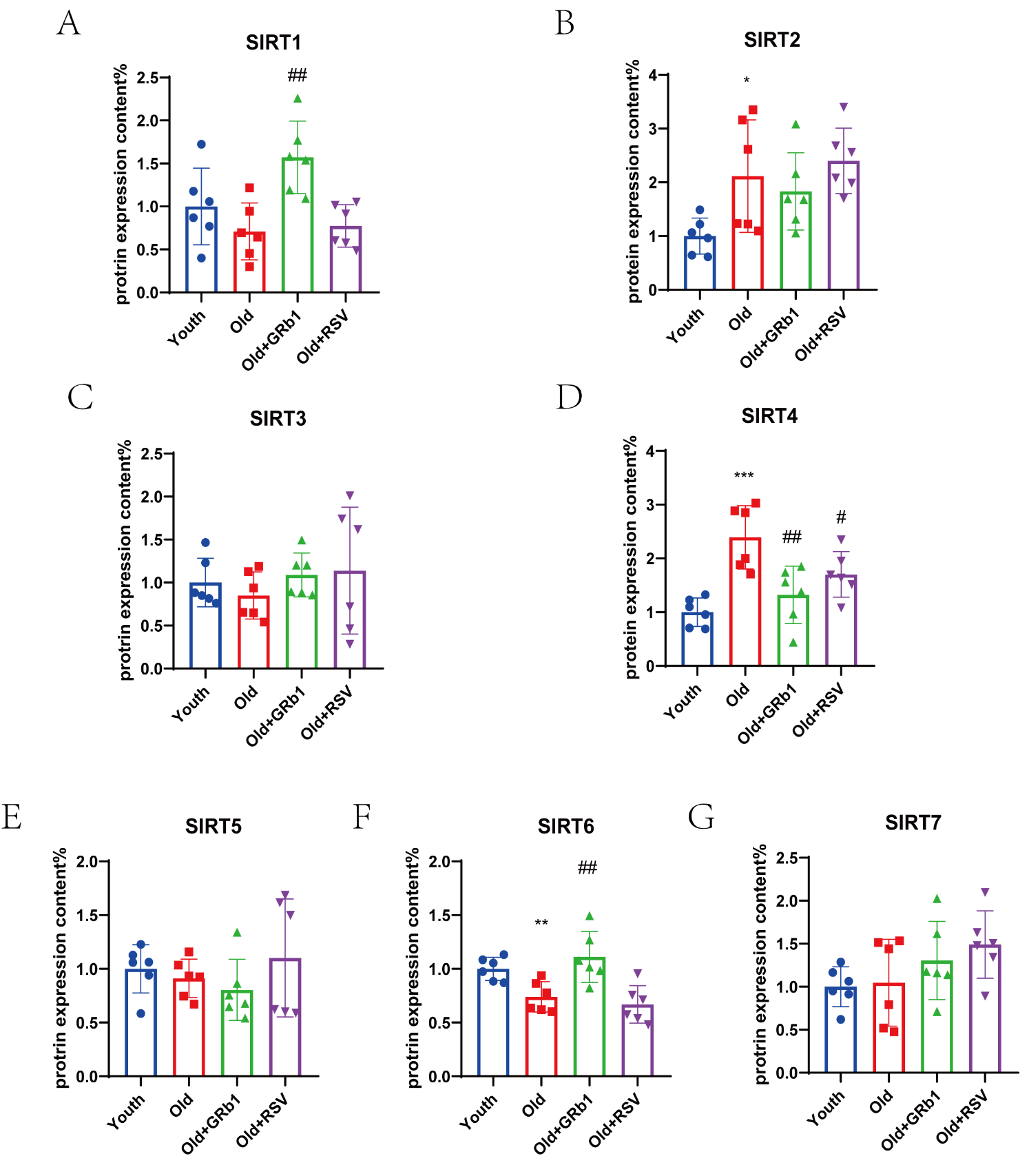


**Supplementary Figure 6. Quantitative Analysis of the Western Blot Results in Figure 4B**

**A-G**. Quantification data of the western blot results of (A) SIRT1, (B) SIRT2, (C) SIRT3, (D) SIRT4, (E) SIRT5 (F) SIRT6 and (G) SIRT7, respectively. 6 mice in each group for 2 times independent experiments. *P<0.05, **P<0.01, ***P<0.001 compared with Youth group; #P<0.05, ##P<0.01 compared with Old group


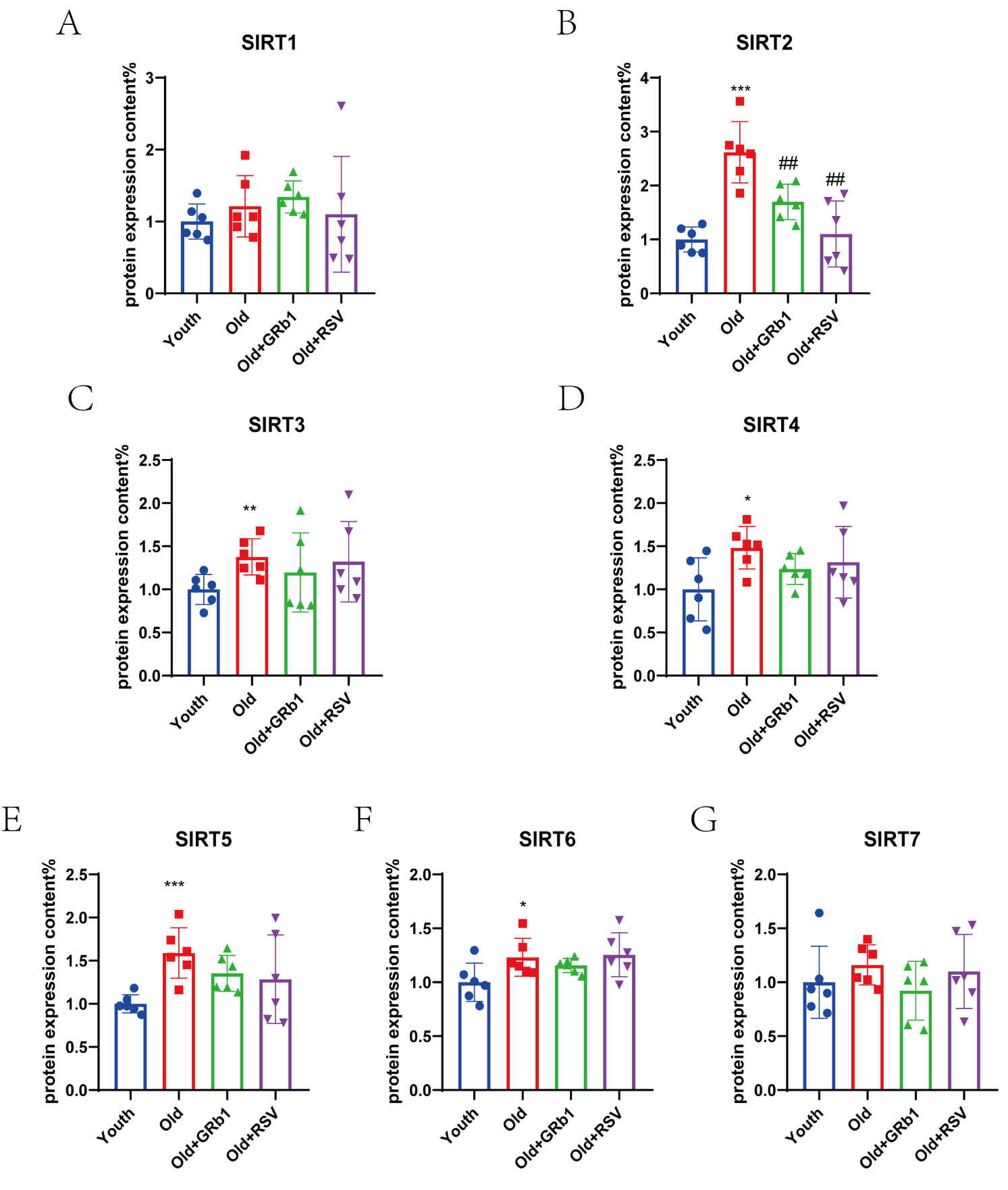


**Supplementary Figure 7. Quantitative Analysis of the Western Blot Results in Figure 4C**

**A-F**. Quantification data of the western blot results of (A) SIRT 1, (B) SIRT 2, (C) SIRT 3, (D) SIRT 4, (E) SIRT 5 (F) SIRT 6 and (G) SIRT 7, respectively. 6 mice in each group for 2 times independent experiments. *P<0.05, **P<0.01, ***P<0.001 compared with Youth group; ##P<0.01 compared with Old group


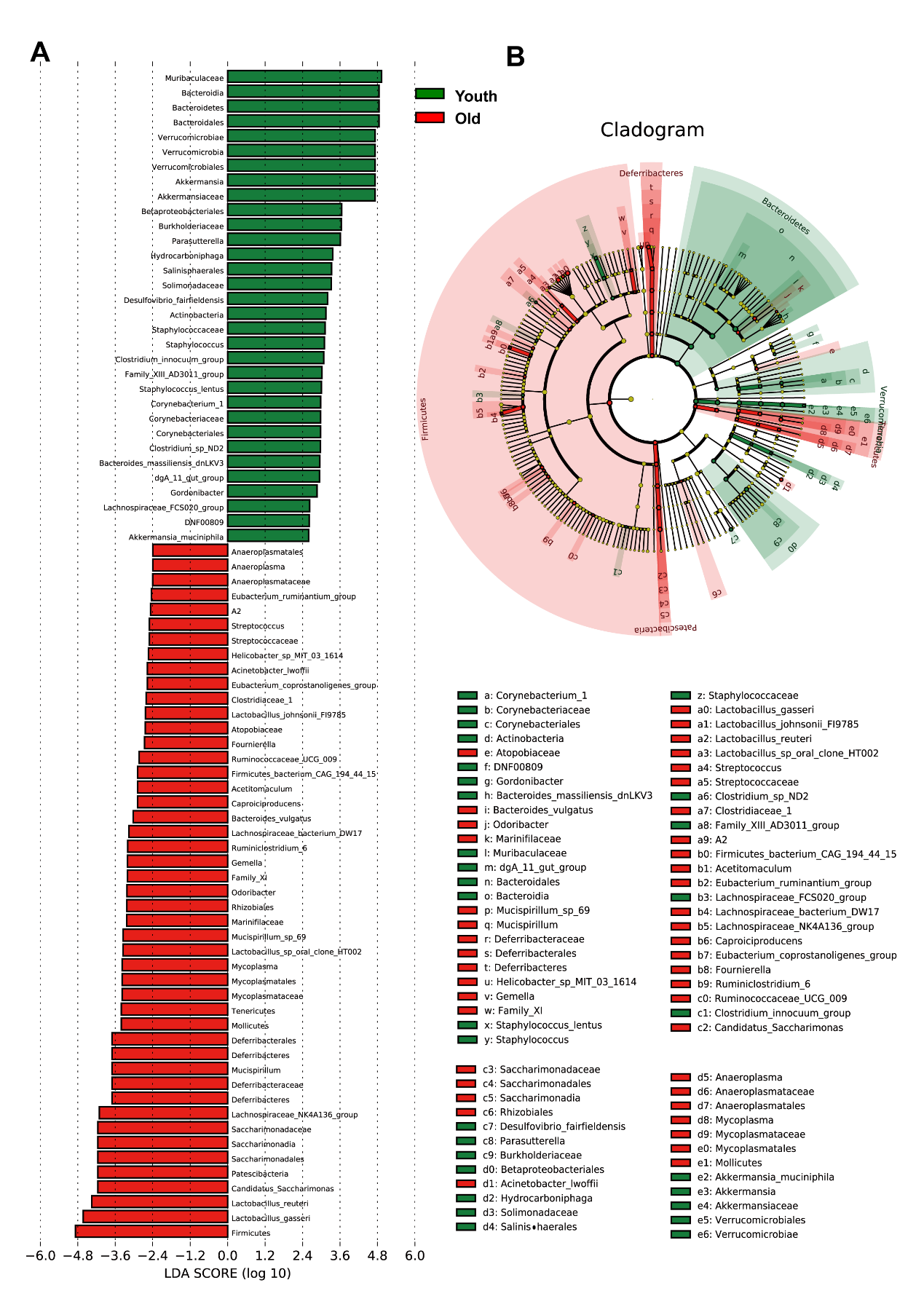


**Supplementary Figure 8. LEFse Analysis Showed the Predominant Taxons for the Youth and Old groups**

**A.** LDA scores showed the predominant abundant taxons for the Youth and Old groups. **B**. Cladogram showed the predominant abundant taxons for the Youth and Old groups. Green represents the bacteria specific for the Youth group, red represents the bacteria specific for the Old group.


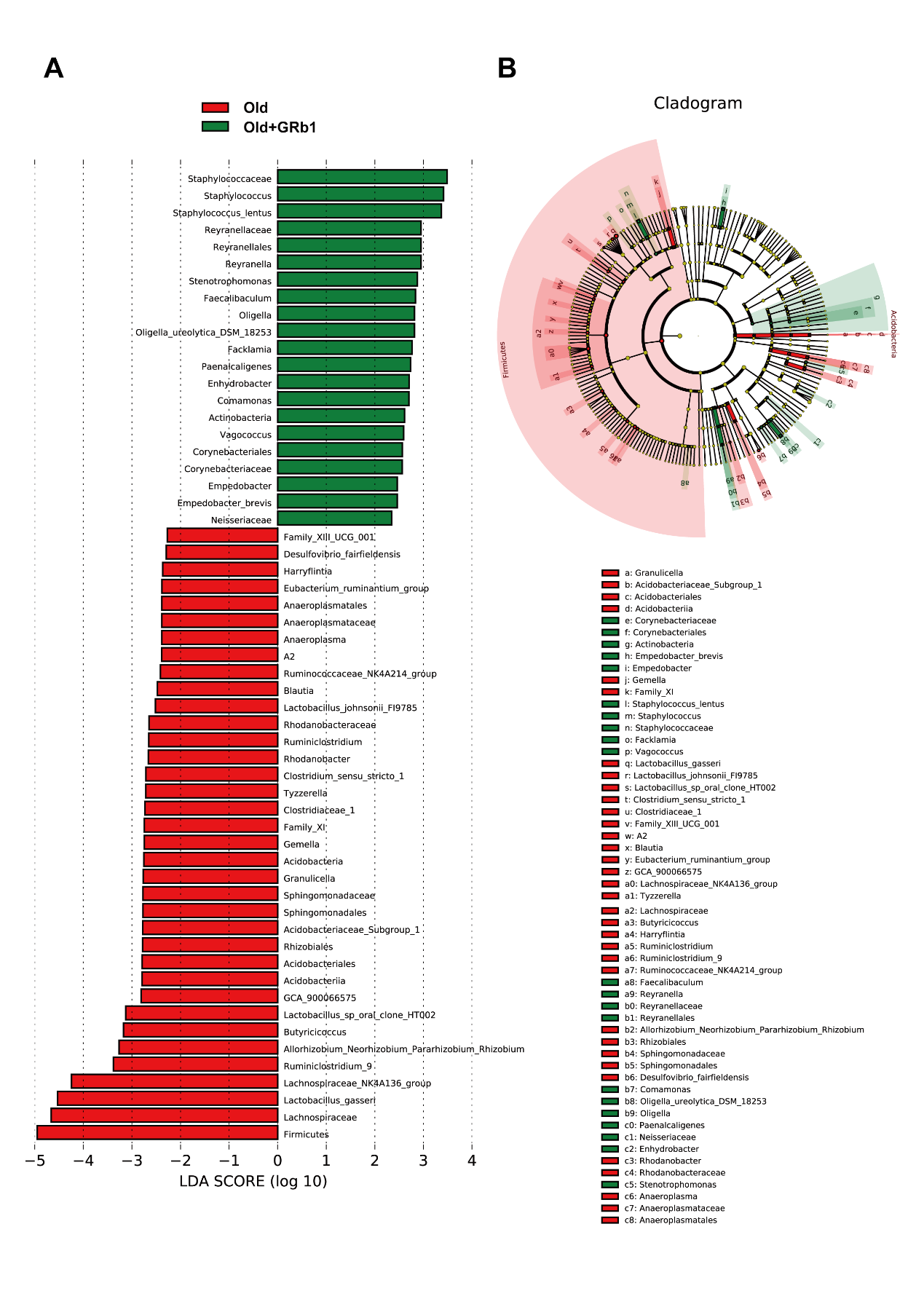


**Supplementary Figure 9. LEFse Analysis Showed the Predominant Taxons for the Old and Old+GRb1 groups**

**A.** LDA scores showed the predominant abundant taxons for the Old and Old+GRb1 groups. **B**. Cladogram showed the predominant abundant taxons for the Old and Old+GRb1 groups. Green represents the bacteria specific for the Old+GRb1 group, red represents the bacteria specific for the Old group. GRb1, Ginsenoside Rb1.


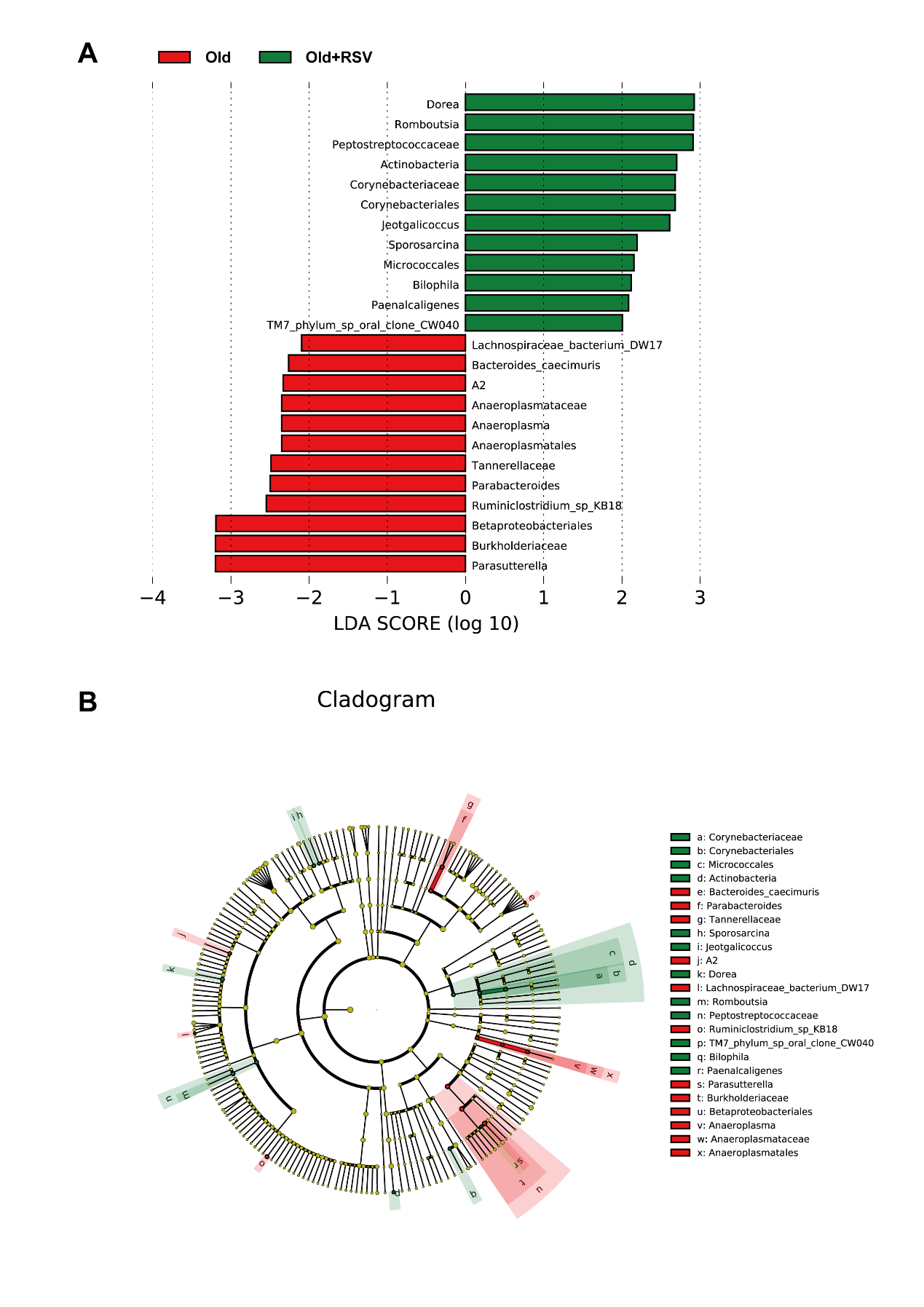


**Supplementary Figure 10. LEFse Analysis Showed the Predominant Taxons for the Old and Old+RSV groups**

**A.** LDA scores showed the predominant abundant taxons for the Old and Old+RSV groups. **B**. Cladogram showed the predominant abundant taxons for the Old and Old+RSV groups. Green represents the bacteria specific for the Old+RSV group, red represents the bacteria specific for the Old group. RSV, Resveratrol.


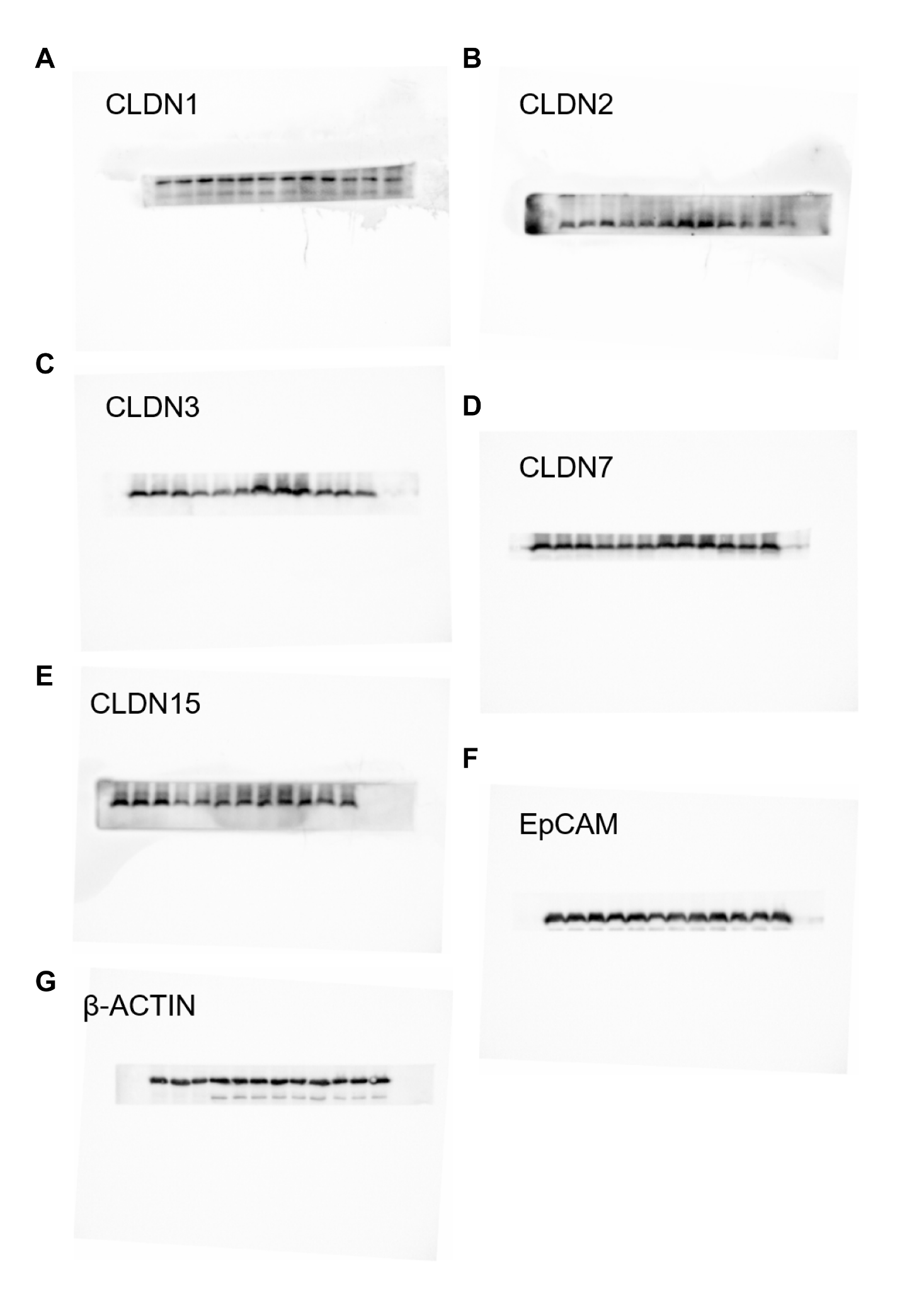


**Supplementary Figure 11. The original western blot bands of Figure 2A were shown**

**A-G.** The original western blot bands in Figure 2A of (A) CLDN1, (B) CLDN2, (C) CLDN3, (D) CLDN7, (E) CLDN15, (F) EpCAM and β-ACTIN respectively.


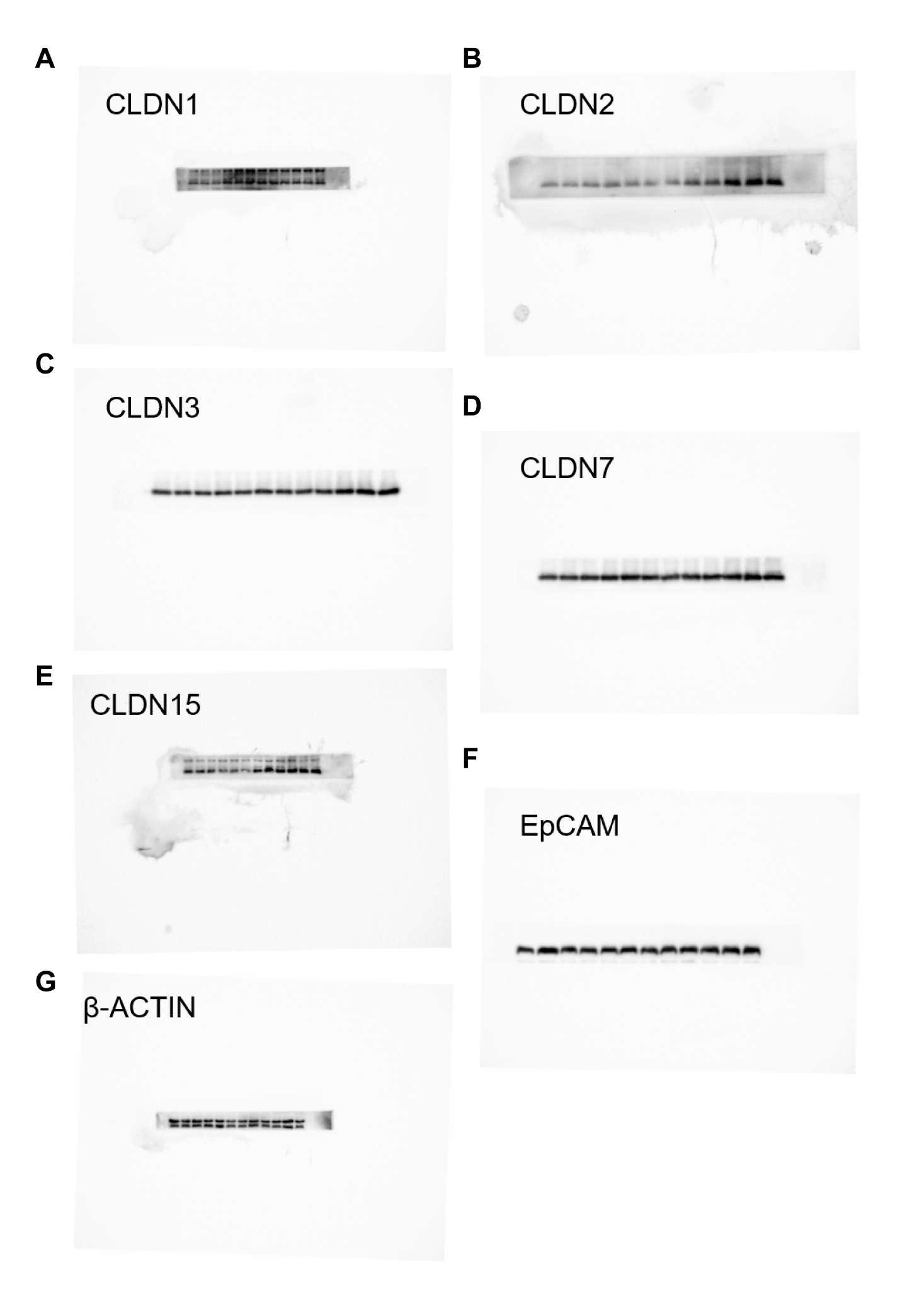


**Supplementary Figure 12. The original western blot bands of Figure 2B were shown**

**A-G.** The original western blot bands in Figure 2B of (A) CLDN1, (B) CLDN2, (C) CLDN3, (D) CLDN7, (E) CLDN15, (F) EpCAM and (G) β-ACTIN respectively.


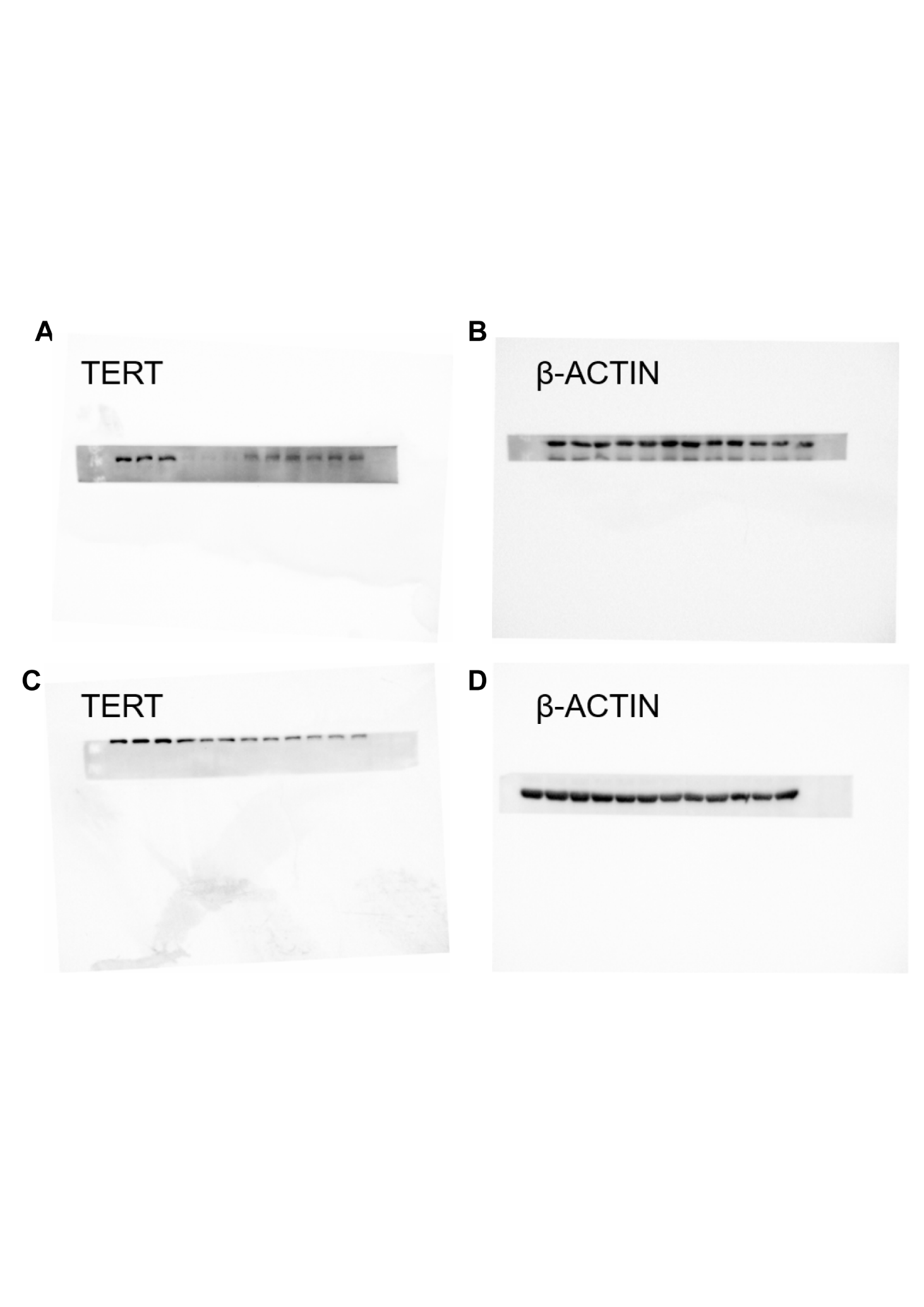


**Supplementary Figure 13. The original western blot bands of Figure 3B-C were shown**

**A-B.** The original western blot bands in Figure 3B of (A) TERT and (B)β-ACTIN respectively. **C-D.** The original western blot bands in Figure 3C of (C) TERT and (D)β-ACTIN respectively.


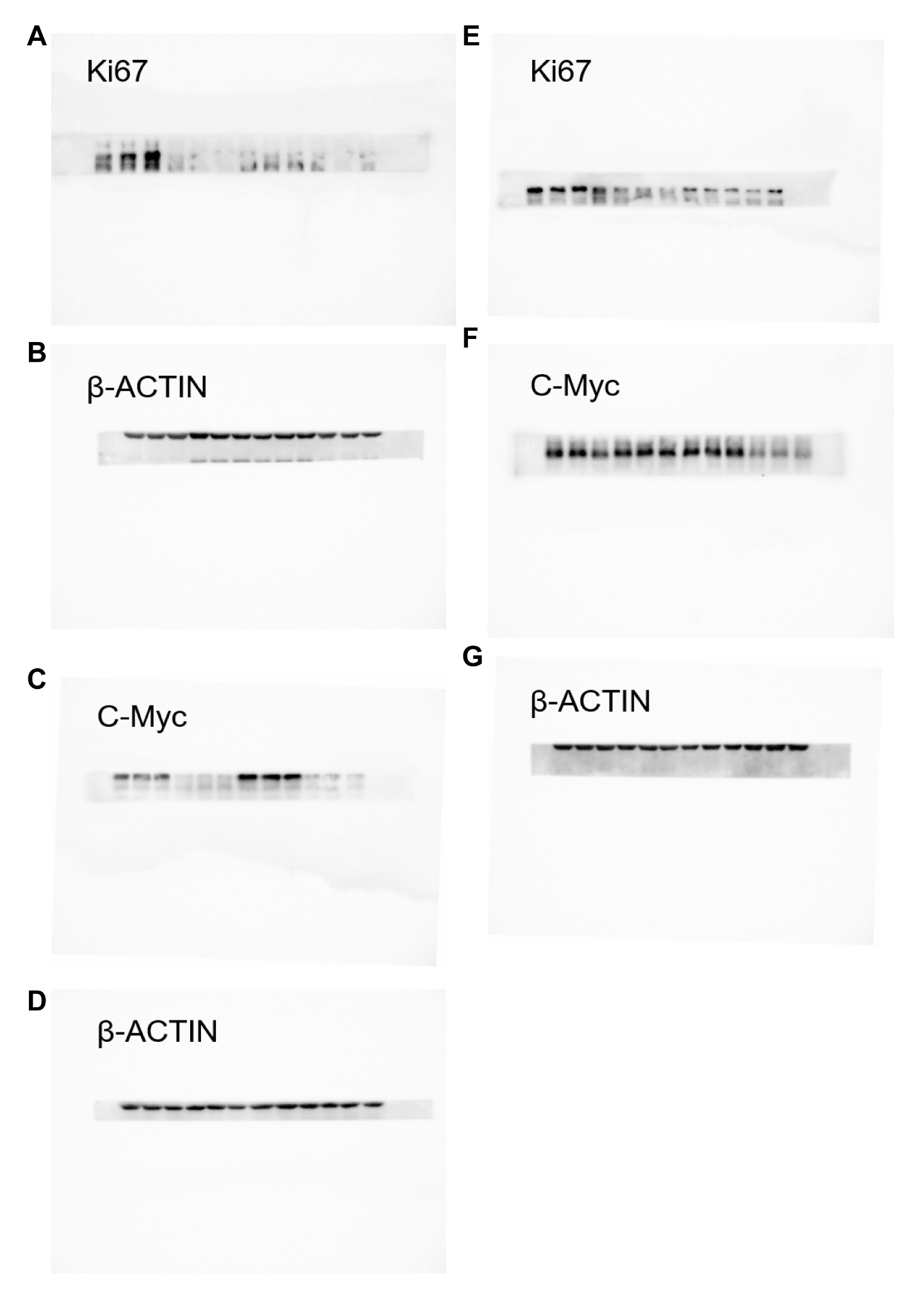


**Supplementary Figure 14. The original western blot bands of Figure 3D-E were shown**

**A-D.** The original western blot bands in Figure 3D of (A) Ki67, (B) β-ACTIN (reference for Ki67), (C) c-Myc and (D)β-ACTIN (reference for c-Myc) respectively. **E-G.** The original western blot bands in Figure 3E of (E) Ki67, (F) c-Myc and (G)β-ACTIN respectively.


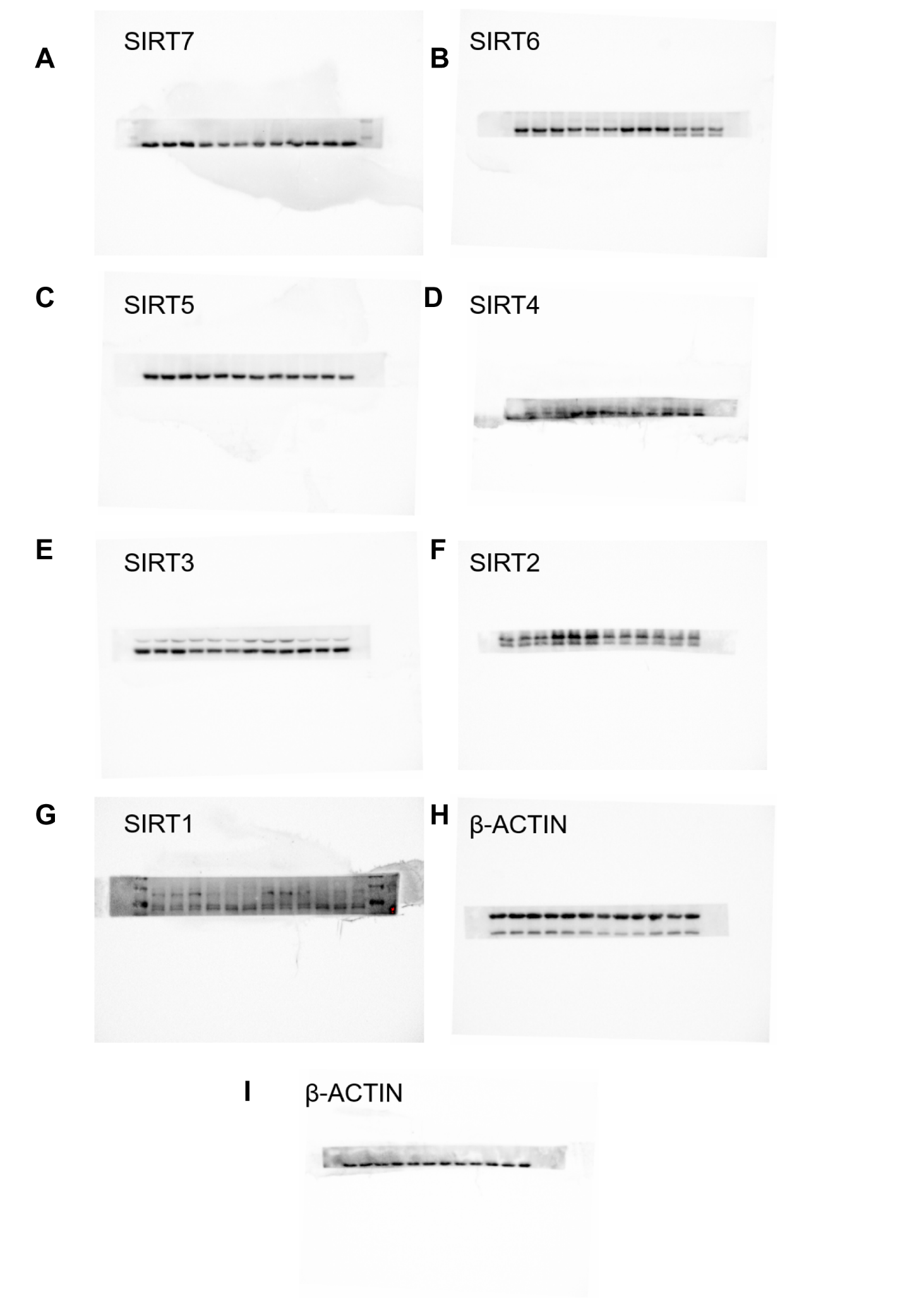


**Supplementary Figure 15. The original western blot bands of Figure 4B were shown**

**A-I.** The original western blot bands in Figure 4B of (A) SIRT7, (B) SIRT6, (C) SIRT5, (D) SIRT4, (E) SIRT3, (F) SIRT2, (G) SIRT1, (H) β-ACTIN for SIRT1, SIRT3, SIRT5, SIRT6, SIRT7 and (I) β-ACTIN for SIRT2 and SIRT4, respectively.


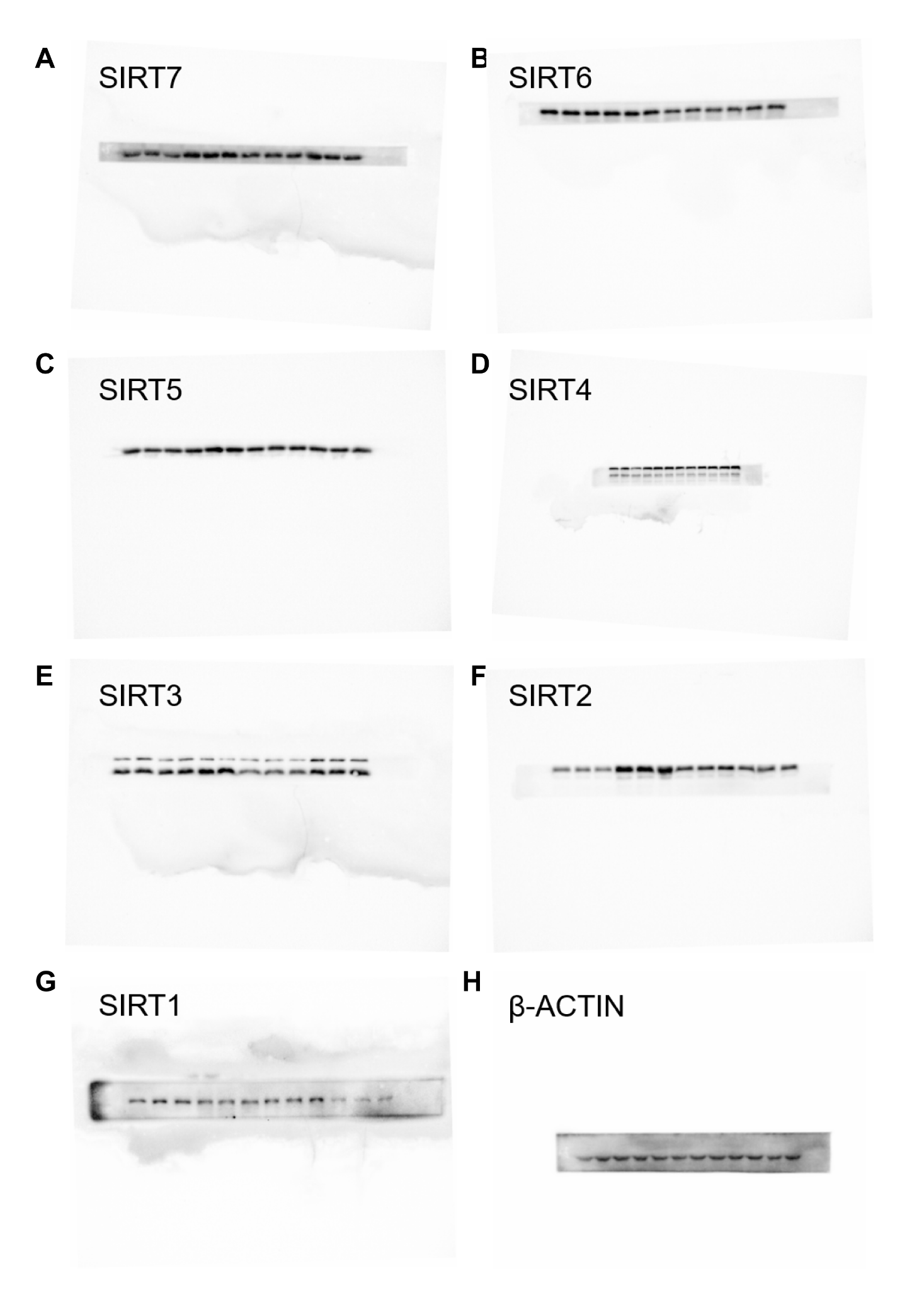


**Supplementary Figure 16. The original western blot bands of Figure 4C were shown**

**A-H.** The original western blot bands in Figure 4C of (A) SIRT7, (B) SIRT6, (C) SIRT5, (D) SIRT4, (E) SIRT3, (F) SIRT2, (G) SIRT1 and (H) β-ACTIN respectively.

**Table S1. The primary and secondary antibodies for Immunofluorescence staining**

**
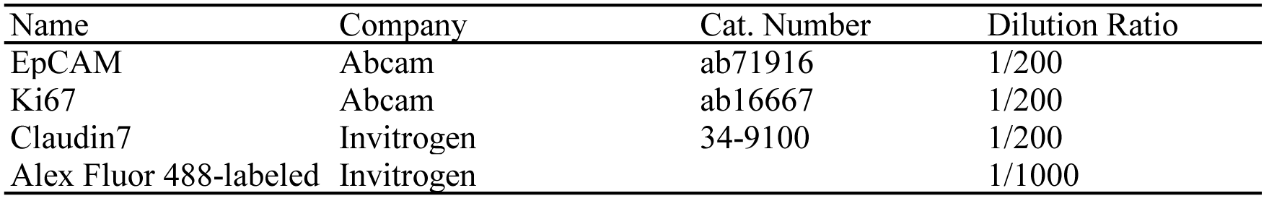
Table S2. Sequences of primers used for qPCR**

**
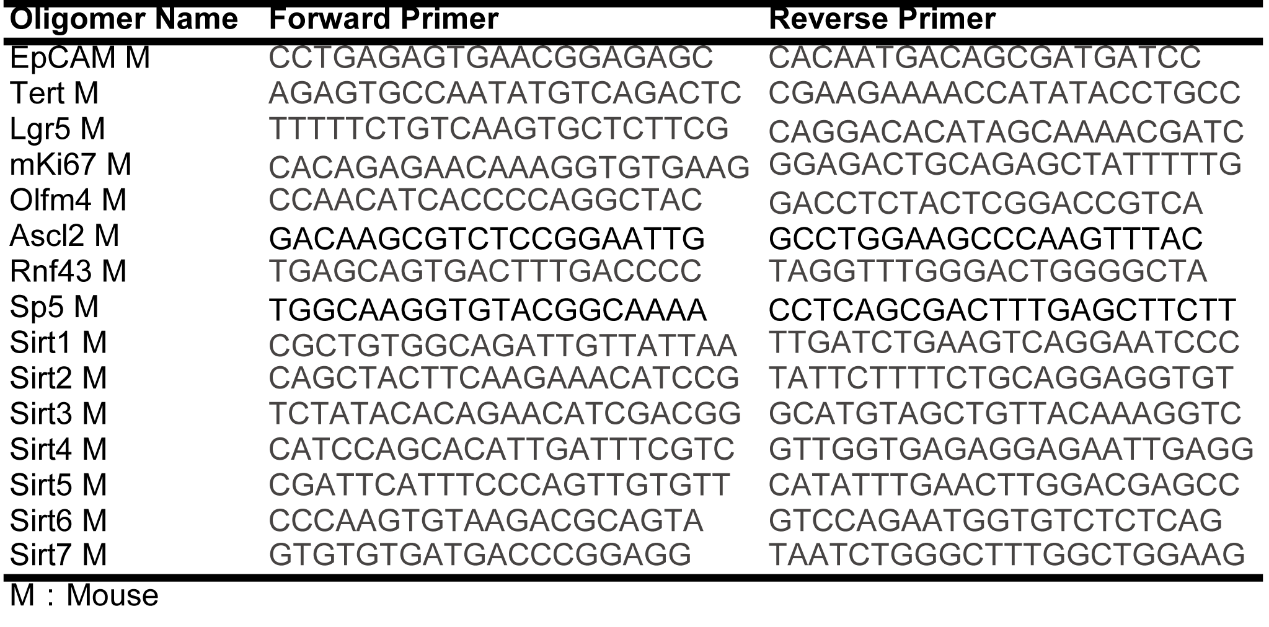
**

**Table S3. The primary antibodies for Western blot**

**
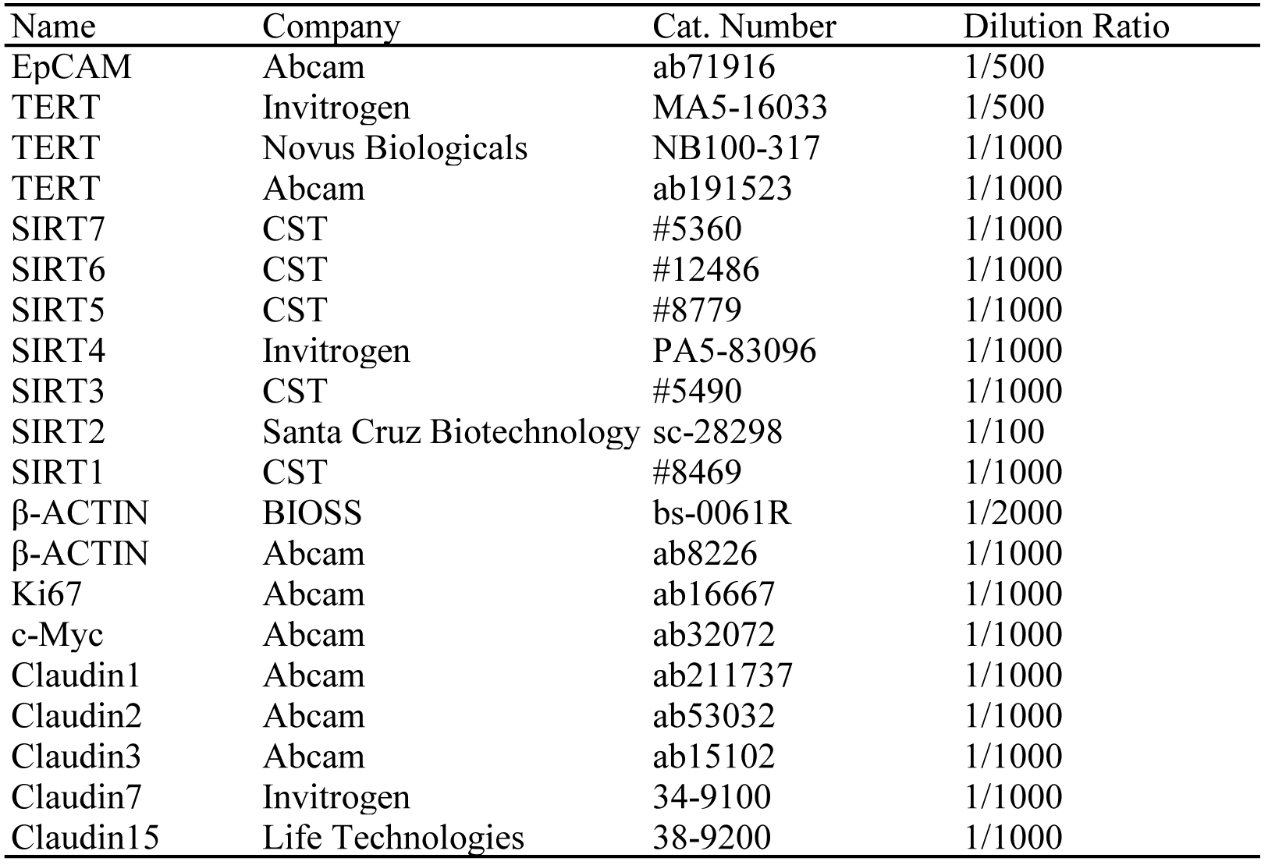
**
